# Honeydew: A Fluorescent Aptamer for Low‐Cost Azo Food Dyes

**DOI:** 10.1002/cbic.70471

**Published:** 2026-07-26

**Authors:** Joseph M. Heili, Paul H. Mroch, Tanner G. Hoog, Orion M. Venero, Grace M. Gustafson, Katarzyna P. Adamala, Aaron E. Engelhart

**Affiliations:** ^1^ Department of Genetics, Cell Biology and Development University of Minnesota Minneapolis Minnesota USA; ^2^ Department of Biochemistry, Molecular Biology and Biophysics University of Minnesota Minneapolis Minnesota USA

**Keywords:** aptamer, fluorescent imaging, RNA

## Abstract

Light‐up RNA aptamers that bind nonfluorescent dyes and conditionally activate their fluorescence are powerful tools for RNA imaging and biomolecular sensing. A barrier to widespread adoption of this technology is a reliance on bespoke dyes prepared by labor‐intensive or costly custom synthesis. Here, we demonstrate that azo dyes—abundant, ultra‐low‐cost (<$1/g), and low‐toxicity molecules widely used in the food and textile industries—can serve as conditionally fluorescent aptamer ligands. We selected aptamers against tartrazine, a ubiquitous azo food dye, and identified a fluorescence‐activating aptamer, which we term Honeydew. We show that Honeydew bound to tartrazine enables real‐time sensing and imaging of model synthetic cells. Additionally, Honeydew selectively activates conditional fluorescence of several other azo dyes, underscoring the untapped potential of this broad dye class. By repurposing ubiquitous industrial dyes, Honeydew is a first‐in‐class fluorogenic azo dye aptamer that provides a highly accessible platform for imaging and sensing applications.

## Graphical abstract

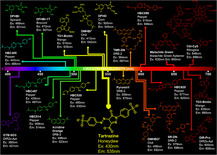

## 1 Introduction

Proteins—the primary catalytic and functional polymers in biology—are readily monitored by fluorescence due to the existence of a large suite of fluorescent proteins that work well in virtually any organismal chassis. Essentially every protein in biology is made through the agency of RNA—rRNA reads out and synthesizes protein, employing tRNAs to decipher codons on mRNA templates. Yet fluorescent monitoring of RNA has proven more difficult than doing the same for proteins. This is because, at the moment, we lack examples of natural fluorescent RNAs with high brightness and visible excitation and emission lines. Fusions of RNA‐binding viral coat proteins with fluorescent proteins like GFP are commonly used to image RNA [1]. This is challenging because fluorescent proteins are intrinsically fluorescent and can produce high background signals. Imaging protein fusions that direct localization to overcome background signals can also be difficult to deploy. This is because they can redirect localization of their client RNAs, exhibit their own maturation kinetics, and can oligomerize themselves [2, 3, 4, 5, 6, 7, 8]. Genetically encoded labels made of RNA could solve some of these problems, and this has resulted in a great deal of interest in RNA tags in recent years.

Numerous groups have selected high‐brightness fluorescent aptamers by selecting for RNAs that bind dyes with low intrinsic fluorescence (due to fast nonradiative decay) but high fluorescence potential (obtained when nonradiative decay is suppressed). The most common form of this strategy involves dyes with a photoisomerizable double bond within a conjugated system. Selections have yielded RNAs with complex tertiary structures that fold into functional ligand‐binding conformations to bind conditionally fluorogenic small molecules via hydrogen bonding, hydrophobic and stacking interactions, and electrostatic complementarity [9, 10, 11, 12, 13, 14, 15, 16, 17, 18]. Nearly all of these aptamers employ bespoke dyes requiring custom synthesis. We sought to make a new aptamer that binds tartrazine, a widely available, inexpensive, FDA‐approved (as tartrazine) azo food dye.

## 2 Results

### 2.1 Tartrazine Exhibits Favorable Properties for Aptamer‐Induced Fluorescence

**FIGURE 1:**
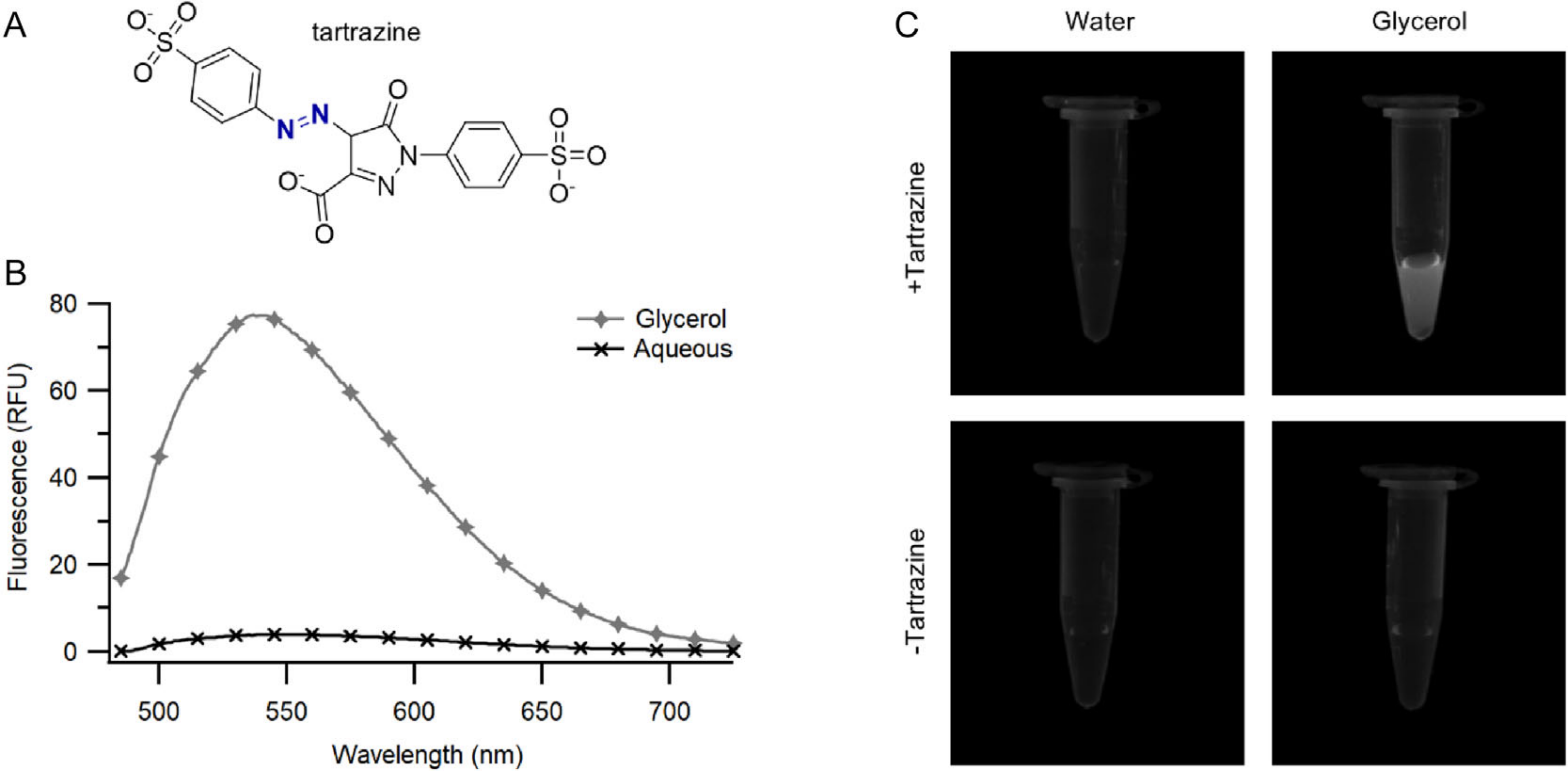
Viscosity‐induced fluorescence enhancement of tartrazine. (A) Chemical structure of tartrazine with the central azo linkage highlighted in blue. (B) Tartrazine (50 μM) emission spectra in 90% glycerol (diamonds) versus aqueous selection buffer (crosses). Sparse markers used for labeling. (C) Fluorescence imaging of 10 μM tartrazine (top) and no‐dye controls (bottom) in water (left column) versus glycerol (right column). Images were acquired under identical settings under blue epi‐illumination (460–490 nm) with an orange emission filter.

Many fluorescent aptamers mimic GFP by binding nonfluorescent ligands in a way that reduces nonradiative excited‐state decay. Tartrazine contains a conjugated system bridged by an azo linkage. We speculated that this structural feature was a significant contributor to tartrazine's low fluorescence quantum yield (Figure 1A), and that the double bond enabled nonradiative decay pathways that could be suppressed by a viscous microenvironment. We tested this by comparing ligand fluorescence in water (low viscosity) versus glycerol (high viscosity) [19, 20]. Tartrazine exhibited ca. 20‐fold fluorescence enhancement in 90% glycerol versus aqueous solution, demonstrating its potential as a conditionally fluorescent ligand (Figures 1B and S1) with favorable properties for aptamer‐induced fluorescence enhancement. Notably, the quantum yield of free tartrazine (5.9 × 10^−5^) is exceptionally low relative to other common aptamer ligands (cf., e.g., DFHBI‐1T, with a quantum yield of 9.8 × 10^−3^) (Table 1). The viscosity of 90% glycerol is 219 cP at 20 °C (ca. 218‐fold higher than water) [31]. To probe whether glycerol‐dependent fluorescence enhancement was principally viscosity‐mediated (vs., e.g., polarity), we evaluated the fluorescence enhancement in water, glycerol, ethanol, methanol, and acetonitrile (Figure S1). At room temperature only glycerol (the most viscous solvent tested) significantly activated tartrazine fluorescence. When frozen in liquid nitrogen (i.e., 77 K, below the freezing point of all solvents used), all samples gave strong emission, consistent with fluorescence enhancement by a viscous or glassy microenvironment.

**TABLE 1:** Characteristics of commonly used fluorescent RNA aptamer systems.

	**Ex./Abs. max** a **, nm**	**Em. max** b **, nm**	Stokes shift, nm	**ε,** **M** ^**−1**^ **cm** ^**−1**^	**QY** d **, ϕ**	Brightness	Kd, nM	**T**_**50%**_ **(relative to 24 °C)** c
Tartrazine^o^	425	550	125	2.68 × 10^4^	(5.9 ± 0.82) × 10^−5^	1.58	n/a	n/a
Honeydew/Tartrazine^o^	430	535	105	2.12 × 10^4^	(1.5 ± 0.064) × 10^−3^	31.8	110	38
Malachite green dye^e^	618	628^f^	10	n/a	7.9 × 10^−5^	n/a	n/a	n/a
Malachite green aptamer^e^	630	655	25	1.5 × 10^5^	0.187	2.8 × 10^4^	117	65^g^
DFHBI‐1T^h^	426	495	69	n/a	9.8 × 10^−4^	n/a	n/a	n/a
Spinach2/DFHBI‐1T^h^	482	505	23	3.1 × 10^4^	0.94	2.9 × 10^4^	560	39^i^
Broccoli/DFHBI‐1T^i^	472	507	35	2.96 × 10^4^	0.94	2.78 × 10^4^	360	50
Pepper/HBC530^j^	485	530	45	6.53 × 10^4^	0.66	4.3 × 10^4^	3.5	57
Mango‐I^k^	510	535	25	7.75 × 10^4^	0.14	1.0 × 10^4^	3.2	44^l^
Mango‐IV^l^	510	535	25	7.75 × 10^4^	0.41	3.2 × 10^4^	11.1	45
DMHBI+^m^	493	540	47	n/a	n/a	n/a	n/a	n/a
Chili/DMHBI+^m^	413	542	129	2.1 × 10^4^	0.4	8.2 × 10^3^	63	42
DFHO^n^	473	561	88	1.98 × 10^4^	6.0 x 10^−4^	11.9	n/a	n/a
Corn/DFHO^n^	505	545	40	2.9 × 10^4^	0.25	7.25 × 10^4^	70	44

a Absorbance or excitation maximum.

b Emission maximum.

c Temperature at which aptamer‐ligand complexes exhibit half the fluorescent signal observed at 24 °C.

d Quantum Yield.

e [21].

f [22].

g [23].

h [24].

i [25].

j [26].

k [27].

l [28].

m [29].

n [13].

o [30].

### 2.2 Multiple Aptamers Selected Against Tartrazine Exhibit Fluorescence Enhancement

**FIGURE 2:**
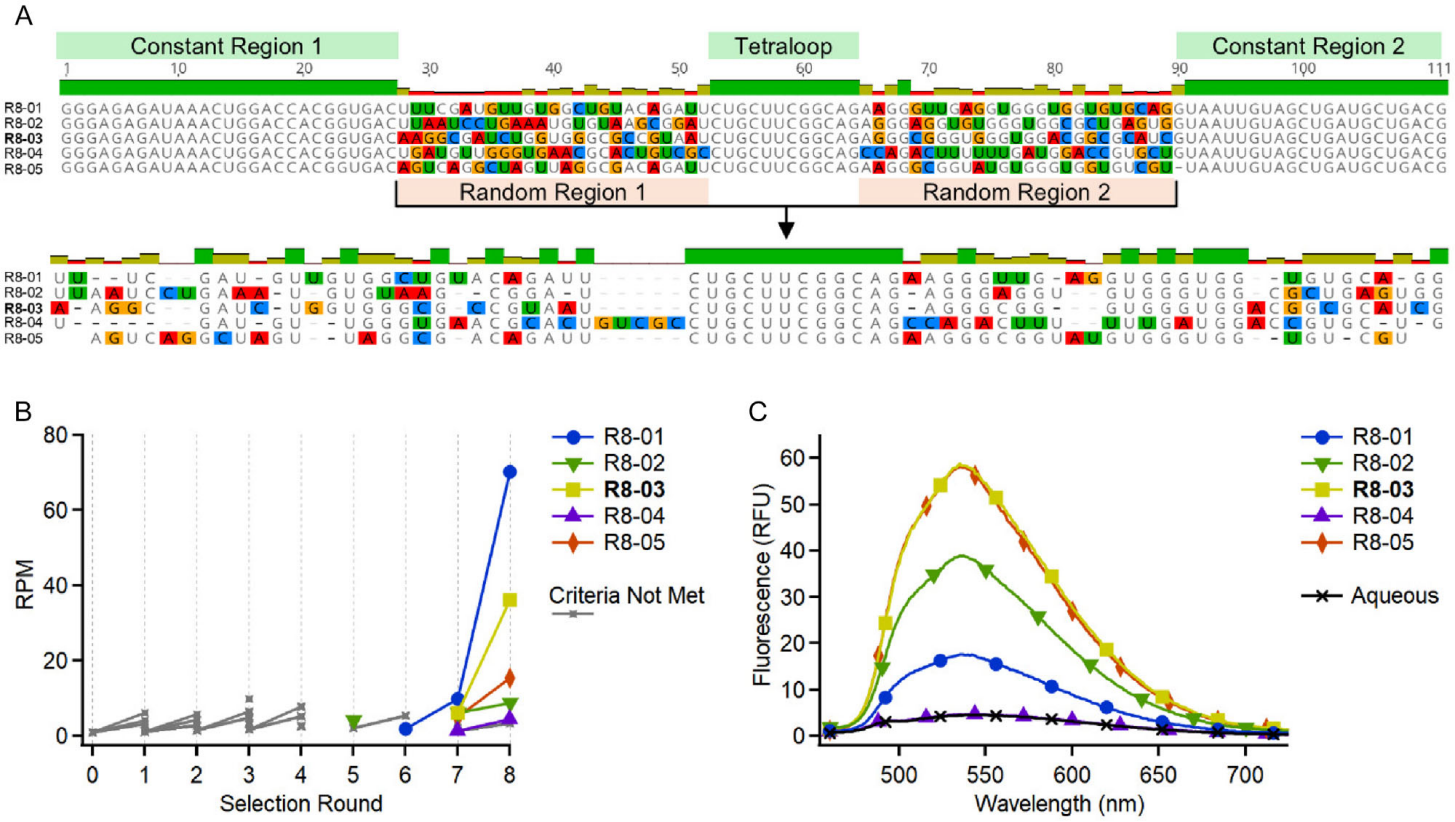
SELEX aptamer candidates. (A) Sequences of the initial aptamer candidates identified via sequencing analysis after eight rounds of selection, with Honeydew sequence identifier (R8‐03) bolded. Inset shows alignment with gaps of the randomized library region excluding the flanking primer binding sites. Alignment via Geneious version 10.2. (B) Graph depicting the enrichment of sequences detected after eight rounds of selection. Data shown tracks the RPM per round of the top 200 sequences when sorted by enrichment score after round 8. Sequences that did not meet our criteria for characterization are depicted in gray while the top five aptamer candidates are shown to have significant enrichment and are present in the round 8 library. (C) Emission spectra of tartrazine dye in aqueous solution with candidate aptamers. Sparse markers used for labeling.

Using Systematic Evolution of Ligands by Exponential Enrichment (SELEX), we selected RNA aptamers against tartrazine. Briefly, library members were annealed in the presence of tartrazine agarose and eluted with free tartrazine (Methods). By round 8 (R8) of the selection, several enriched sequences emerged. We screened five R8 sequences for enhancement of tartrazine fluorescence, based on an RPM enrichment‐based criteria (Supporting Methods, Figure S2). Of these, 4 of 5 showed significant fluorescent signal under excitation at 420 nm (Figure 2). We selected sequence R8‐03 for further characterization—terming it Honeydew. This sequence was selected because it was among the most fluorescence‐enhancing sequences, while also having the second highest sequence enrichment score in our selection. We additionally used a nonfluorescent (1.02‐fold fluorescence enhancement) sequence, R8‐04, as a control sequence for subsequent experiments.

**FIGURE 3:**
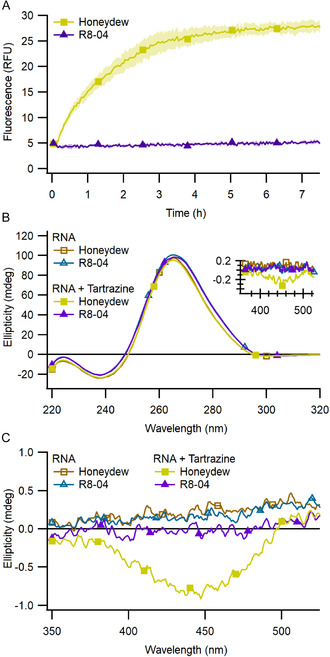
Fluorescence‐monitored in vitro transcription and steady‐state circular dichroism of Honeydew and R8‐04 in the presence of tartrazine. (A) Transcription of Honeydew and R8‐04 in the presence of 100 μM tartrazine shows increased fluorescence upon transcription of Honeydew, but not R8‐04. Sparse markers are used as labels, shaded area represents S.E.M., *n* = 3. (B) Circular dichroism of Honeydew and R8‐04 with 25 μM RNA, 62.5 μM tartrazine in fluorescence assay buffer. Visible region shown in inset. Sparse markers are used as labels. (C) Circular dichroism visible region of Honeydew and R8‐04 with 100 μM RNA, 250 μM tartrazine. Sparse markers are used as labels.

**FIGURE 4:**
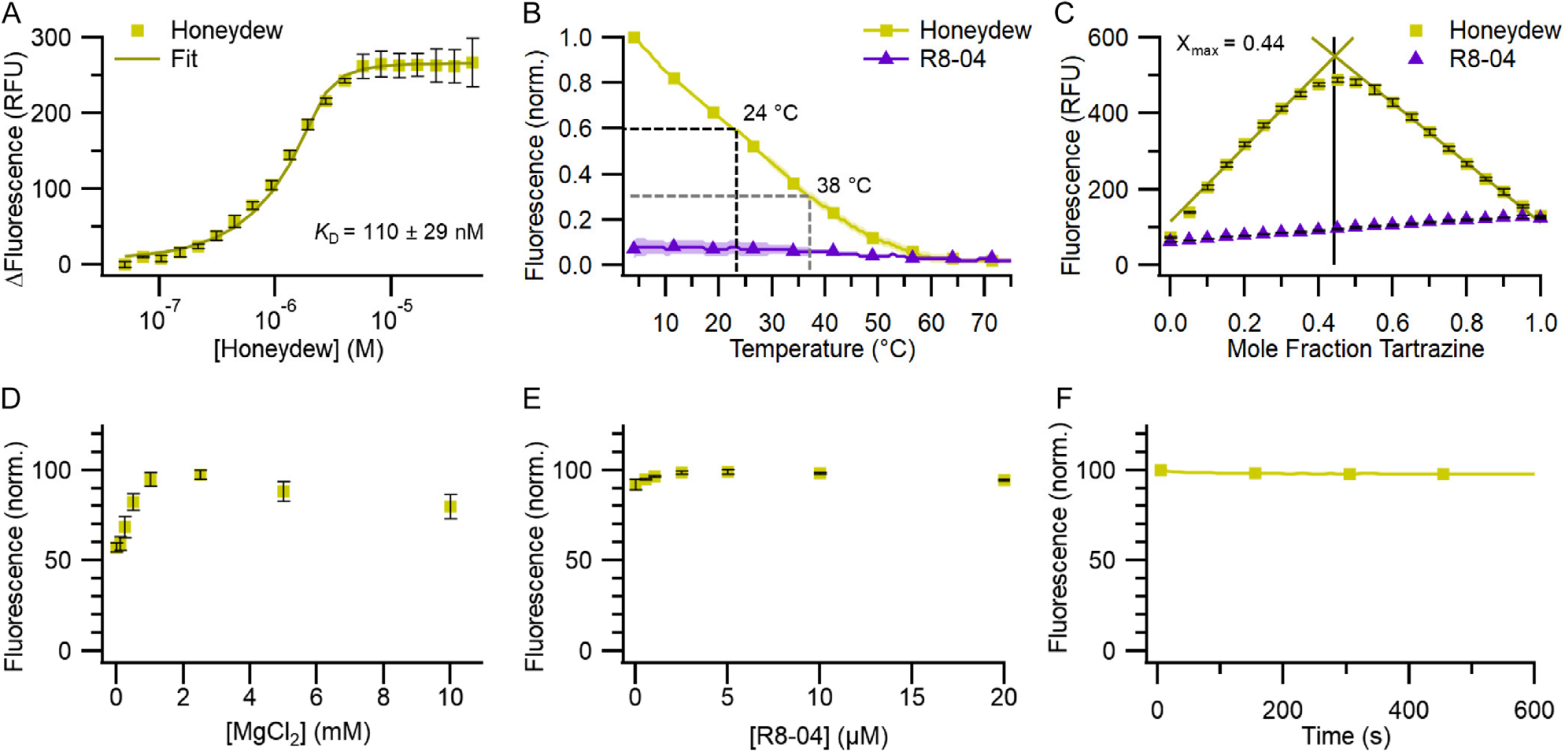
Characterization of the binding interaction between Honeydew and tartrazine. (A) Fluorescence‐monitored dissociation constant for Honeydew–tartrazine complex. [Honeydew] was held constant at 2.5 µM. Emission data were blanked relative to tartrazine‐free RNA in fluorescence assay buffer. Error bars represent S.E.M., *n* = 3. (B) Fluorescence‐monitored thermal denaturation of Honeydew and R8‐04 in the presence of tartrazine. The curve shown in each case was the second heating trace following an initial heat‐cool cycle measured at the same heating rate as the data shown. Data were normalized to the maximum and minimum fluorescence values for both conditions. Shaded area represents S.E.M., *n* = 3. Sparse markers are used as labels. (C) Job plot for stoichiometry determination of Honeydew–tartrazine (yellow) and R8‐04‐tartrazine (purple) interactions. Bars represent S.E.M., *n* = 3. (D) Magnesium dependence of Honeydew–tartrazine complex measured via fluorescence spectroscopy. Data were normalized to the highest fluorescence reading. Error bars represent S.E.M., *n* = 3. (E) Fluorescence‐monitored tartrazine (5 µM) binding competition between Honeydew (5 µM) and R8‐04. Data were normalized to the highest fluorescence reading. Error bars represent S.E.M., *n* = 3. (F) Fluorescence‐monitored photostability of Honeydew–tartrazine complex collected under continuous excitation. Data were normalized to *t* = 0 values. Shaded area represents S.E.M., *n* = 3. Sparse markers are used as labels.

We next used the tartrazine–Honeydew complex for fluorescent monitoring of in vitro transcription (Figure 3A). Transcription of Honeydew in the presence of tartrazine gave increasing fluorescence signal over ca. 3 h, while the inactive sequence R8‐04 showed no change in fluorescence, despite a comparable isolated yield of RNA product upon purification (Figure S3). Operating Honeydew–tartrazine complex in the presence of increasing [R8‐04] did not suppress Honeydew–tartrazine fluorescence up to the highest [R8‐04] tested (fourfold molar excess), consistent with R8‐04 not effectively outcompeting Honeydew for tartrazine binding (Figure 4E).

To further demonstrate tartrazine binding by Honeydew aptamer, we obtained circular dichroism spectra for Honeydew and R8‐04 in the presence and absence of tartrazine (Figure 3B). Both sequences exhibited a strong positive peak at 265 nm and a slight dip at 238 nm, with a slightly narrower peak width for Honeydew versus R8‐04, consistent with a different fold. Honeydew–tartrazine complex additionally exhibited a small negative induced band at ca. 450 nm, consistent with tartrazine (*λ*_max_ = 430 nm) bound in a chiral microenvironment. This signal increased further at fourfold higher RNA and tartrazine concentration (Figure 3C). At both concentrations, only the Honeydew–tartrazine complex exhibited an induced band, indicating a selective Honeydew‐tartrazine‐like interaction did not occur with R8‐04.

### 2.3 Honeydew is a High‐Affinity Aptamer to Tartrazine

We measured the affinity of the Honeydew–tartrazine complex by fluorescence‐monitored titration of tartrazine in the presence of constant [RNA]. Honeydew–tartrazine exhibited an apparent *K*_D_ of 110 nM (Figure 4A), and R8‐04 exhibited no fluorescence‐observed interaction with tartrazine. Upon addition of Honeydew to tartrazine, maximum fluorescence occurred within the ∼4 s sample mixing period, demonstrating rapid complex formation (Figure S5). We additionally examined the absorbance spectrum of tartrazine in the presence and absence of Honeydew. Honeydew caused a 5 nm redshift in the absorbance spectrum of tartrazine, consistent with a change in electronic microenvironment (Figure S6).

The thermal response of the fluorescent signal of the Honeydew–tartrazine complex resembles that of other fluorescent RNA aptamers. We did not observe a low‐temperature plateau region, consistent with an overall greater structuring of the binding pocket at low temperature. The fluorescence midpoint relative to room temperature was 38 °C (Figure 4B).

To interrogate the stoichiometry of the binding interaction between Honeydew and tartrazine, we used Job's method of continuous variation to monitor fluorescence at varying aptamer:ligand stoichiometries with [aptamer + ligand] held constant (yellow squares, Figure 4C). This revealed a ca. 1:1 binding stoichiometry. R8‐04 (purple triangles, Figure 4C) showed no defined‐stoichiometry RNA–tartrazine interaction. Though Honeydew–tartrazine fluorescence is relatively insensitive to magnesium concentration, we observed maximum fluorescence between 1 and 2.5 mM MgCl_2_ (Figure 4D). To probe the photostability of the Honeydew–tartrazine complex, we measured its emission during an illumination period of 10 min. More than 97% of the fluorescent signal was maintained throughout the experiment (Figure 4F). We additionally truncated the aptamer and found a ca. 60 nt minimal binding region (Figure S4).

### 2.4 Azo Dyes Cross‐Affinity Enables Multispectral Emission from Honeydew

One feature of fluorescent aptamers that distinguishes them from proteins is that they bind an extrinsic ligand, enabling use of the same aptamer for measurements at different wavelengths. We reasoned that azo food dyes, which share a diaryldiazene core, might also bind Honeydew. This has been observed in other fluorescent aptamer systems, such as Spinach, Pepper, SRB‐2, and Clivia, which bind multiple members of a dye class [24, 32, 33, 34].

**FIGURE 5:**
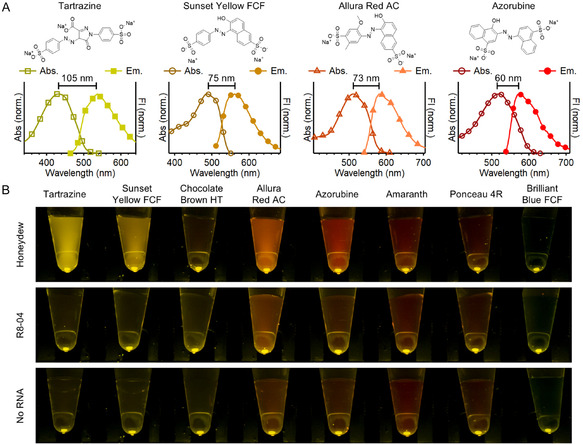
Honeydew enhances the fluorescence of multiple azo food dyes. Absorbance/fluorescence spectroscopy of Honeydew (20 μM) and fluorescence photography of Honeydew and R8‐04 (20 μM) in the presence of azo food dyes (10 μM). (A) Normalized absorbance and emission spectra of azo dyes in the presence of Honeydew. Sparse markers are used as labels. (B) Fluorescence photography of tubes containing Honeydew (top image), R8‐04 (middle image), and no added RNA (bottom image) in the presence of specified azo dyes, illuminated under identical conditions with a blue light gel transilluminator and photographed using an orange emission filter. All three images were captured and processed using identical settings.

We screened Honeydew and R8‐04 with seven additional azo dyes: Sunset Yellow FCF, Chocolate Brown HT, Allura Red AC, Azorubine, Amaranth, Ponceau 4R, and Brilliant Blue FCF. We observed Honeydew‐induced fluorescence enhancement for 4 of 8 azo dyes tested (Figure 5), demonstrating selective yet broad recognition of multiple members of this structural class. R8‐04 showed fluorescence enhancement of only Allura Red AC, and this was markedly lower than Honeydew‐Allura Red AC fluorescence (Figure S6). The Honeydew–tartrazine complex exhibited the largest Stokes shift (ca. 110 nm, Figure 5A) among the Honeydew‐dye pairs tested. The other dyes that bind Honeydew (Sunset Yellow FCF, Allura Red AC, and Azorubine) exhibited fluorescence visible by photography (Figure 5B) with large Stokes shifts as well. The dye ensemble enables detectable emission in the full 500–700 nm range. Tartrazine and Sunset Yellow FCF gave the greatest enhancement when these and a broader ensemble of dyes were tested (Figure S7), consistent with a better‐optimized binding pocket for these closely related dyes.

### 2.5 Fluorescence Microscopy of Honeydew in Liposomes

**FIGURE 6:**
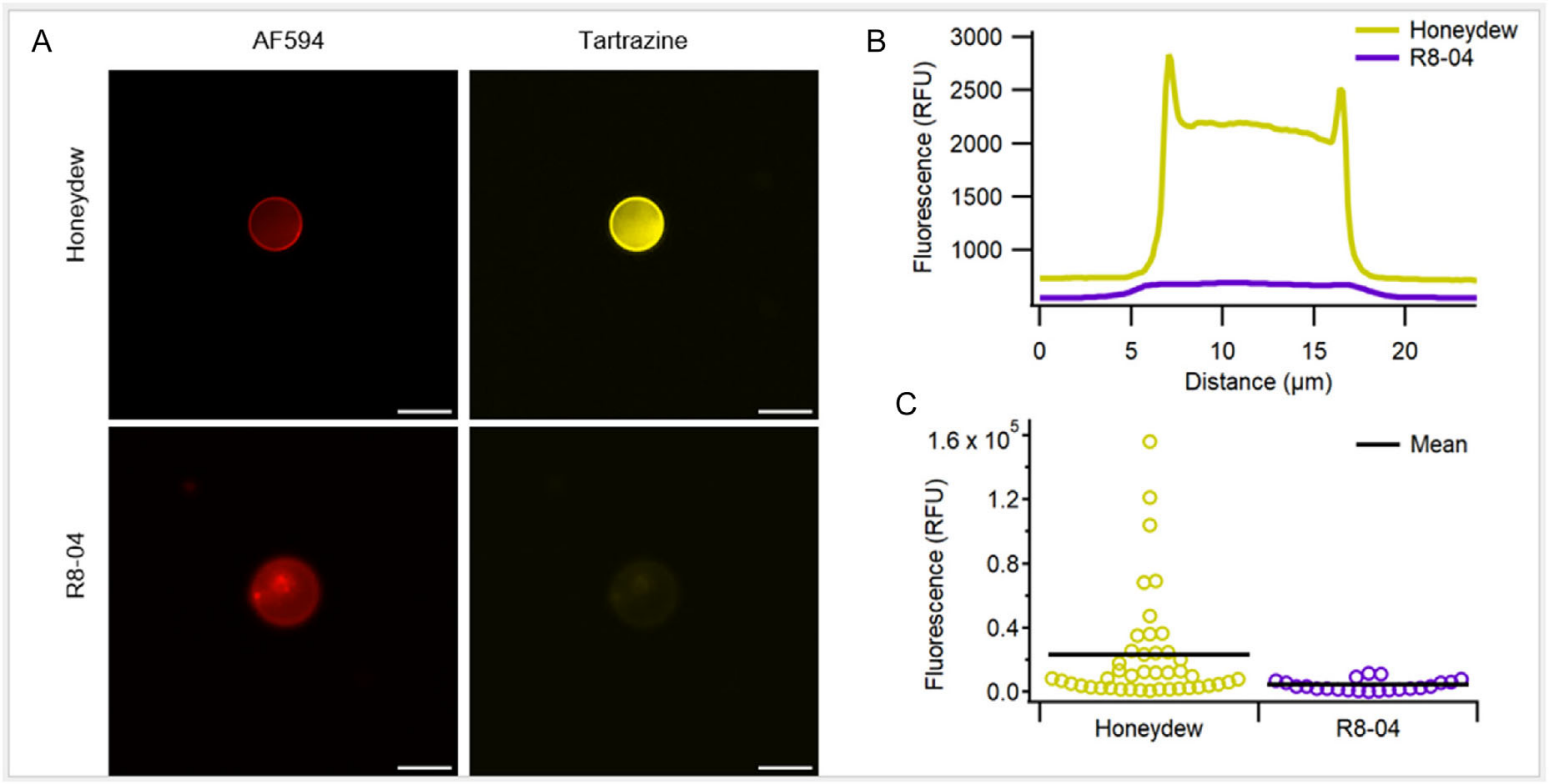
Honeydew enables fluorescence imaging of transcription reactions in model synthetic cells. (A) Representative fluorescence microscopy images of liposomes encapsulating transcription reactions of either Honeydew aptamer (top row) or the R8‐04 negative control RNA (bottom row) in the presence of tartrazine. The left column (membrane dye, AF594) serves as a spatial marker to identify and confirm liposome integrity (Figures S8 and S9). The right column (lumen dye, tartrazine) captures the fluorescence signal of tartrazine. Scale bars 10 μm. (B) Cross‐sectional spatial profile of the tartazine fluorescence channel across the representative liposomes depicted in (A). (C) Beeswarm plot displaying the population‐level quantification of tartrazine fluorescence across all captured fields of view for Honeydew transcribing liposomes (yellow circles, *n* = 43) and the R8‐04 transcribing liposomes (purple circles, *n* = 22). Black horizontal lines denote the mean fluorescence values for each population.

The low cost of Honeydew makes it an ideal aptamer system for enablement of the design‐build‐test cycle in synthetic biology and synthetic cell research. To evaluate the imaging performance of Honeydew in a cell‐like environment, we encapsulated transcription machinery within liposomes and transcribed Honeydew. Using AF594‐labeled lipids, we confirmed successful liposome formation and structural integrity (Figures 6A, S8 and S9). Upon incubation at 37 °C, liposomes transcribing Honeydew exhibited fluorescence signal visible by fluorescence microscopy (Figure 6B). This signal was significantly higher than that of the liposomes transcribing R8‐04 RNA. Quantification across multiple fields of view confirmed this (Figure 6C), underscoring the efficacy of Honeydew as a sensor. Honeydew showed a mean lumen fluorescence 5.3 × higher than R8‐04 (2.2 × 10^4^ RFU vs. 4.3 × 10^3^ RFU, respectively).

## 3 Conclusions

Honeydew is a first‐in‐class light‐up RNA aptamer that binds and activates an azo food dye—a class of ligands notable for having exceptionally low intrinsic background fluorescence. It offers lower cost relative to commonly used aptamers; even malachite green aptamer [21, 35], known for being inexpensive yet cytotoxic [36], is more expensive.

To our knowledge, Honeydew is currently the most cost‐effective fluorescent RNA aptamer system available. Tartrazine is significantly less expensive than bespoke fluorogenic ligands. Other established aptamer ligands, some of which have costs of 4–7 orders of magnitude more than tartrazine, can impose financial barriers to scalability or implementation as read‐outs in preliminary optimization of techniques/technologies (Table 2). We have previously employed light‐up RNA aptamers as fluorescent read‐outs to probe the characteristics of liposomes, namely investigating vesicle formation, membrane leakage, programed fusion, and peptide‐mediated cargo translocation across synthetic cell membranes [37, 38]. While effective, the design and scale of such assays are fundamentally constrained by the prohibitive cost of bespoke ligands; deploying Honeydew enables execution of these experiments at a fraction of the cost.

**TABLE 2:** Price of commercially available conditionally fluorescent ligands for commonly used light‐up RNA aptamer systems.

Ligand	Aptamer	Source	Price ($) per gram
Tartrazine	Honeydew	Fisher Scientific, AAA1768214	0.41
Malachite Green	MG aptamer	Fisher Scientific, AAA1618614	1.33
Sulforhoramine B	SRB‐2	Sigma‐Aldrich, 230 162	25.8
DFHBI‐1T	Broccoli	Fisher Scientific, 561 010	3.67 × 10^4^
TO1‐3PEG‐biotin	Mango	ABM, G7955	2.00 × 10^7^
HBC530	Pepper	Selleckchem, E0078	3.98 × 10^4^
HBC620	Pepper	Selleckchem, E0623	6.18 × 10^4^

Azo dyes are a structurally related class of dyes containing aromatic systems bridged by an azo linkage. The ability of Honeydew to activate the fluorescence of some structurally related azo dyes indicates Honeydew‐class aptamers can bind and activate multiple ligands of this class. This parallels several other aptamers, such as Pepper, which binds diverse HBC derivatives [32], Clivia/Corn/Spinach/Broccoli/Squash, which bind GFP‐like fluorophores [25, 33, 39, 40, 41] and SRB‐2, which binds a family of sulforhodamine B derivatives [34].

While nucleic acid and synthetic systems have previously been developed to interact with azo linkage‐containing compounds, such as light‐controlled riboswitches responsive to azobenzene derivatives [42] and molecularly imprinted non‐nucleic acid polymers used for environmental remediation of azo dyes [43, 44], these systems do not function as fluorescent reporters. To our knowledge, Honeydew represents the first reported RNA aptamer capable of directly binding and activating the fluorescence of an azo dye, establishing a new class of cost‐effective, noncovalent fluorogenic sensors.

The cross‐azo dye reactivity with preference for tartrazine and the related sunset yellow suggests the possibility of selecting pan‐azo dye aptamers, as well as more selective Honeydew derivatives. The absorbance of Allura Red AC and Azorubine align with the Honeydew–tartrazine emission spectrum, offering the potential for establishing a redshifted genetically encoded FRET system extending the already high spectral separation of Honeydew–tartrazine even further.

Honeydew establishes a baseline for future optimization of azo dye‐based light‐up RNA aptamers, by techniques such as cell‐sorting based reselection [25, 41] or mutation library selection and direct fluorescence‐based variant screening using microfluidic compartment sorting methods [45, 46, 47, 48]. These methods have generated high‐performance aptamers in the TO‐derivative‐binding Mango family [49] and HBI‐derivative binding Broccoli [25] and Corn [10, 13, 50]. Directed evolution has been used to generate fluorescent aptamers from parent sequences that target different ligands, as well—the Squash aptamer campaign evolved the adenine riboswitch into a fluorescent aptamer [41, 51]. Furthermore, there have been efforts to improve first‐generation aptamer systems through optimization of the ligands specifically, such as with the Chili aptamer [52]. Honeydew enables an exceptionally low‐cost route to these optimization strategies by binding dyes with over a century of demonstrated low toxicity and in vivo use.

## 4 Materials and Methods

### 4.1 Reagents and Equipment

#### 4.1.1 Oligonucleotides

Unless otherwise stated, all synthetic oligonucleotides were purchased from Integrated DNA Technologies (Coralville, IA) with standard desalting. DNA PCR amplifications were carried out using OneTaq Hot Start 2X Master Mix with standard buffer (NEB, M0484L) in a T100 thermal cycler (Bio‐Rad). Transcription and purification of RNAs performed with adaptations from previously published methods with T7 RNA polymerase were expressed and purified in‐house [53]. Namely, the transcription buffer consisted of 40 mM Tris pH 7.9, 24 mM MgCl_2_, 2 mM sperimidine, 1 mM DTT, 8 mM GTP, 4 mM each of ATP, UTP, and CTP, 1 μM T7 RNAP, 0.4 units/μL RNase Inhibitor Murine (NEB, M0314L), and 0.02 units/μl inorganic pyrophosphatase (Milipore Sigma, I5907). Reverse transcription was performed with SuperScript IV (Thermo Fisher Scientific, 18 090 200). Column‐based nucleic acid purifications used a Monarch Spin RNA Cleanup Kit (NEB, T2050L) with subsequent desalting with PD‐10 columns packed with Sephadex G‐25 resin (Cytivia, 17 085 101). DNA template digestion during RNA purification used TURBO DNAse (Invitrogen, AM2238) according to the manufacturer's protocol. Concentration of RNA or DNA via ethanol precipitation was performed as described previously [54]. Gel purification of oligonucleotides was performed in denaturing conditions on 20 cm × 22 cm × 0.8 mm 10% Urea‐PAGE gels run for 2.5 h at 12 W, followed by visualization for gel excision using TLC plate UV shadowing. Nucleic acid was recovered from the gel by band excision, extrusion of gel slice through a 25 ga needle with 3 mL 1 × TBE, and 3 X freeze–thaw cycles in liquid nitrogen and a 60 °C water bath. Following a 1 h final elution at room temperature, the supernatant was removed by centrifugal filtration. Purified RNAs were refolded by an initial 5‐min incubation at 75 °C followed by cooling at 5 °C increments for 2 min intervals, with a 5 °C final temperature.

#### 4.1.2 Dyes

Azo dyes often have multiple names referring to the same dye. The names of the dyes used as ordered from the vendor were as follows: Acid Yellow 23 (Tartrazine) (TCI, F0088), Sunset Yellow FCF (TCI, S0141), Allura Red AC (TCI, A0943), Azo Rubine (Carmosine) (TCI, A0580), Acid Blue 9 (Brilliant Blue FCF) (TCI, B0790), Acid Red 27 (Amaranth, Azorubine) (TCI, A0583), Acid Red 18 (Ponceau 4R) (TCI, N0309), Bromophenol Blue (VWR, CAYM14331‐50), Coomassie Brilliant Blue G‐250 (GoldBio, C‐460‐25), Chlorophenol Red (Millipore Sigma, 199 524‐10G), Xylene Cyanol FF (Millipore Sigma, X4126‐10G), and DFHBI‐1T (Tocris, 5610).

#### 4.1.3 Preparation of Affinity Matrix

Affinity resin was generated using EAH Sepharose 4B (Cytiva, 7 056 901) with EDC (Milipore Sigma, 39 391) and tartrazine. EAH Sepharose 4B was washed with 25 volumes each of DI H_2_O, 500 mM NaCl, and DI H_2_O. A 20 × molar excess of tartrazine relative to resin amine sites was coupled using the EAH Sepharose 4B manufacturer instructions with adaptations, and this protocol was carried out two times in succession. Specifically, coupling was carried out overnight at room temperature in the dark using 200 mM EDAC (N‐(3‐Dimethylaminopropyl)‐N′‐ethylcarbodiimide hydrochloride, Oakwood Chemical Item No. 024810) and 100 mM sulfo‐NHS (N‐Hydroxysulfosuccinimide, Cayman Chemical Item No. 20 680), adjusted to pH 5 with LiOH. After the second coupling reaction, unreacted amine sites on the resin were capped using 100 mM acetate (added as acetic acid), 200 mM EDAC, and 100 mM Sulfo‐NHS, adjusted to pH 5 with LiOH. The resin was then washed with 25 volumes each of DI H_2_O, 0.1 M lithium acetate pH 4.0, 0.1 M Tris‐HCL pH 8.0 with 500 mM LiCl, and DI H_2_O. The resulting tartrazine‐sepharose was stored in 20% ethanol. Immediately prior to use in selection the affinity matrix was washed with 500 volumes each of DI H_2_O, 500 mM LiCl, DI H_2_O, followed by three washes with 25 volumes 1X selection buffer (40 mM Li‐HEPES pH 7.4, 125 mM LiCl, 5 mM MgCl_2_). A fully acetylated counterselection resin was also prepared by capping unfunctionalized resin with acetic acid/EDAC/sulfo‐NHS as described in the final capping step for tartrazine resin.

#### 4.1.4 Library Design and Amplification

A 4 nmol (ca. 2 × 10^15^ sequence) library [40, 55] was ordered from Integrated DNA Technologies (Coralville, IA) with standard desalting. The library consisted of an internal conserved 12‐base tetraloop‐stem to seed RNA structure elements, flanked by 26‐base random regions and 5′ and 3′ primer binding sites (Table S1). PCR amplification was used to create dsDNA transcription template and the 5′ forward primer was designed with an overhang to install the 5′ T7 RNAP recognition sequence. To reduce potential amplification artifacts associated with amplification of random libraries, PCR conditions were optimized for only 12 amplification cycles. A high volume (38.4 mL aliquoted into 96 well plates and later pooled) of PCR reaction mix consisting of 10 nM template and 0.2 µM primers underwent denaturing at 94 °C, annealing at 53 °C, and elongation at 68 °C. PCR products were treated with Exonuclease I (New England Biolabs M0293S) to digest primers, concentrated using ethanol precipitation, and resuspended in 8 M Urea 1 X TBE Buffer for gel loading for purification using 10% Urea‐PAGE gel. Finally, another ethanol precipitation was used to concentrate the gel purified DNA library prior to transcription. RNA library was generated via in vitro T7 RNAP transcription of 1.64 nmol of dsDNA in a total transcription volume of 6.3 mL. Purification of the RNA transcripts was done by template dsDNA digestion, Monarch spin column purification, and Sephadex gravity column desalting. Following purification, RNA polymers were annealed. After each round of selection, RNAs eluted from selection resin were reverse transcribed, cDNA was amplified via PCR with full length product isolated using denaturing PAGE gel electrophoresis purification, and RNA was generated by T7 RNAP transcription for input into the subsequent selection round.

#### 4.1.5 Spectroscopy

Absorbance spectra were recorded at 24 °C with an Agilent Cary 60 (Agilent Technologies) using quartz 3 mm × 3 mm cuvettes. Fluorescence emission spectra were measured at 24 °C using a Cary Eclipse (Agilent Technologies) and quartz 3 mm × 3 mm cuvettes. Unless otherwise stated, assays for characterization of aptamer candidates were carried out in fluorescence assay buffer consisting of 40 mM Tris pH 7.9, 5 mM MgCl_2_, 100 mM NaCl. Thermal melts and qPCR were carried out on a CFX384 Touch (Bio‐Rad).

#### 4.1.6 SELEX

Purified RNA libraries and selection resins were prepared as described above. Both were suspended in selection buffer consisting of 40 mM lithium‐HEPES pH 7.4, 125 mM LiCl, and 5 mM MgCl_2_. At a ratio of 2:1 counter selection sites to RNA molecules, 500 µL RNA in selection buffer was incubated with functionally capped counter selection resin at 37 °C for 30 min, with pipette mixing every 5 min. Eluted nonbinding RNAs from the counter‐selection resin were collected with an additional 250 µL selection buffer wash and incubated with the tartrazine‐functionalized resin (1:1 ratio of tartrazine sites to RNA molecules) for 30 min at 37 °C with mixing at 5 min intervals. Nonbinders were then washed from the resin with 5 mL of selection buffer with an incubation of 30 min at 37 °C. Tartrazine‐binding sequences were then eluted with four elution steps, 2 × 250 µL 5 mM tartrazine elutions incubated for 30 min at 37 °C, followed by a 250 µL buffer wash (for a total tartrazine elution volume of 750 µL), and 2 × 250 µL urea elution buffer elutions incubated for 30 min at 37 °C, followed by a 250 µL buffer wash (for a total urea elution volume of 750 µL). Urea elution buffer consisted of 8 M urea in 10 mM lithium‐EDTA pH 8. In round 6, competition for binding sites was increased by reducing the ratio of available tartrazine binding sites relative to RNA to 1:2.5 by decreasing the volume of resin and increasing the concentration of RNA. Also in round 6, the stringency was increased by increasing the tartrazine resin wash step volume to 10 mL. For rounds 7 and 8, the ratio of binding sites to RNA was kept the same, but washing volume of nonbinders from the resin was increased to 20 mL of selection buffer, in 5 mL increments with 7.5 min incubation per increment at 37 °C.

#### 4.1.7 Data Analysis

Selection rounds were submitted for NGS (AMPLICON‐EZ, GENEWIZ/Azenta) with partial Illumina adapters installed by PCR. Index addition, sequencing (typically MiSeq, as reported in FASTQ files), and demultiplexing were performed by the sequencing vendor. Sequencing analysis consisted of sequence trimming with cutadapt 5.2 [56] and read quality filtering with FASTX‐Toolkit [57], count and clustering analysis via FASTAptamer 2 [58, 59], and individual sequence enrichment and identification analysis using a HoneydewCountCompiler.py script developed in‐house (Section S2). Sequences were additionally screened with the MEME Suite [60] using the STREME module [61]. Candidate sequences were visualized as secondary structures using the RNAfold module of the ViennaRNA 2.7.0 package [62, 63]. Graphs and curve fits were generated with Igor Pro version 9 (Wavemetrics).

Parsing these sequences based on a number of criteria we chose a subset of ten sequences to screen individually for fluorescence (Figures 2B and S2A). Sequences R8‐01 through R8‐05 were chosen based on RPM and round‐by‐round enrichment criteria (Figure 2B), while R8‐06 through R8‐10 were chosen based on clustering and motif search analysis (Figure S2A).

#### 4.1.8 Fluorescence Emission Spectra Assays

Individual sequences of interest were ordered as dsDNA from IDT. Fluorescence‐monitored transcriptions were carried out with 100 µM tartrazine added to the transcription master mix and observed via SpectraMax Gemini EM microplate reader (Molecular Devices; SoftMax Pro 5.4). Emission spectra for screening of initial aptamer candidates (Figure 2) were collected using 50 µM purified RNAs and 50 µM tartrazine in selection buffer and visualized on a Cary Eclipse fluorescence spectrophotometer (Agilent Technologies) at excitation 420 nm, excitation and emission slits 10 nm, medium PMT (600 V). Under the same conditions and parameters emission of aqueous tartrazine was compared with tartrazine in 90% glycerol.

#### 4.1.9 Circular Dichroism

Circular dichroism spectra were obtained on a J‐815 CD Spectrophotometer (JASCO) using 1 mm pathlength quartz cuvettes. A 1:2.5 ratio of RNA to tartrazine was used so that all RNA was bound to tartrazine. Spectra were collected in a fluorescence assay buffer consisting of 40 mM Tris pH 7.9, 5 mM MgCl_2_, and 100 mM NaCl. Spectra were scanned from 600 to 220 nm with a data pitch of 1 nm, a digital integration time of 2 s, and at a scan rate of 100 nm/min. Data shown are the average of five accumulations.

#### 4.1.10 Affinity Measurements

Dissociation constants (*K*_D_) were calculated by plotting the concentration of tartrazine versus fluorescence signal collected during stepwise dilution of tartrazine in the presence of constant RNA concentration (2.5 µM) in fluorescence assay buffer monitored on a Cary Eclipse (Agilent Technologies). For each tartrazine concentration a corresponding fluorescence reading was collected with no RNA present and used as a baseline blank for that particular dye concentration. Fit curves were determined using the equation: f(*C*_t_)=(((((*C*_t_*K_b_ + *E*_t_*K_b_ + 1)−((*C*_t_*K_b_ + *E*_t_*K_b_ + 1)^2^−(4*(*K*_b_)^2^)*E_t_*C_t_)^0.5^)/(2*K*_b_))*(*F* ^b^−*F* ^0^)/*E*_t_) + *F* ^0^)

where *C*_t_ is the titrated component, *K*_b_ is the association constant, *E*_t_ is the fixed component (here, RNA), *F* ^0^ is fluorescence at dye concentration zero, and *F* ^b^ is fluorescence plateau value at high dye concentration [64].

#### 4.1.11 qPCR Fluorescence Melts

Thermostability of Honeydew and R8‐04 was assessed in excess RNA conditions to prevent interference from unbound dye. 10 µM RNA was mixed with 5 µM tartrazine in fluorescence assay buffer, and fluorescence intensities were recorded using a Bio‐Rad CFX384 Touch in FRET mode (excitation 450–490 nm and emission 560–580 nm) at 0.5 °C increments from 4 to 75 °C with 20 s at each temperature for sample equilibration. Values presented are the mean and S.E.M. from three independent measurements.

#### 4.1.12 Job Plot

Fluorescence was recorded for 21 solutions with varying RNA:tartrazine ratios and total RNA + tartrazine concentration of 15 µM in fluorescence assay buffer. Fluorescence spectra were collected at excitation wavelength 420 nm and average emission from 700–750 nm was used as baseline for each spectra. Job plots were created by plotting the average emission at wavelengths 540 ± 15 nm versus mole fraction of tartrazine.

#### 4.1.13 Magnesium Dependence

The fluorescence of the Honeydew–tartrazine complex was evaluated across varying concentrations of MgCl_2_. Fluorescence spectra of 5 µM RNA and 2.5 µM tartrazine in fluorescence assay buffer were collected on a Cary Eclipse fluorescence spectrophotometer (Agilent Technologies) at excitation 420 nm, excitation and emission slits 10 nm, and PMT 950 V. Spectra were blanked with control samples containing tartrazine but lacking RNA and the maximum peak fluorescence values were recorded.

#### 4.1.14 Honeydew Versus R8‐04 Competitive Binding Assay

Competitive binding for tartrazine (2.5 µM) between Honeydew (5 µM) and various concentrations of R8‐04 by collecting fluorescence spectra using a Cary Eclipse fluorescence spectrophotometer (Agilent Technologies) at excitation 420 nm, excitation and emission slits 10 nm, and PMT 950 V. Spectra were blanked with tartrazine samples lacking RNA and the maximum peak fluorescence values were recorded.

#### 4.1.15 Photostability

Photostability of the Honeydew–tartrazine complex was evaluated using kinetic monitoring of the fluorescence signal for 5 µM Honeydew and 2.5 µM tartrazine in fluorescence assay buffer on a Cary Eclipse fluorescence spectrophotometer (Agilent Technologies) under continuous excitation at 420 nm with excitation slit 20 nm, emission at 540 nm with emission slit 10 nm, PMT 950 V, and with 10 s averaging time per data point for 10 min. All fluorescence values were normalized to the initial *t* = 0 value.

#### 4.1.16 Quantum Yield

Quantum yield measurements for aqueous tartrazine and Honeydew–tartrazine complex were determined using quinacrine (Φ = 0.075) [65] as an actinometer by calculating the slope of plots with the integral of the emission spectra versus the absorbance collected at the excitation wavelength for a range of sample concentrations. Measurements for RNA‐fluorophore complexes were collected under conditions of excess RNA to eliminate background effects of unbound fluorophore. Three replicates were collected for each of two distinct experimental conditions (total *n* = 6) with final quantum yields reported as the grand mean with standard error of the independent determinations.

#### 4.1.17 Specificity Screening of Azo Food Dyes

Various azo food dyes were screened for fluorescent interaction with Honeydew and R8‐04 using an in‐house imaging rig with subsequent collection of absorbance spectra and fluorescence spectra at the determined excitation maxima. For each sample, 20 µM RNA was mixed with 10 µM dye in fluorescence assay buffer to ensure that all dye present was bound. Tubes were imaged on a 3D‐printed rig using a blue light gel transilluminator (Denville, 1 005 860) as the light source. Images were collected using a Sony a6500 camera with SEL50M28 lens using the orange transilluminator filter, exposure time 30 sec, ISO 320, f/4, white balance set to fluorescent, and postprocessing of RAW images using Camera Raw 12.0 PS with adobe color profile, temp 3800, tint 21, exposure +1.55, highlight +5, and blacks −59.

Samples were then transferred to 3 mm × 3 mm quartz cuvettes for collection of absorbance spectra, and fluorescence spectra were collected with fluorescence assay buffer used as a blank. Fluorescence assay parameters in the Cary Eclipse fluorescence spectrophotometer (Agilent Technologies) were as follows: Tartrazine: excitation 428 nm, ex slit 10 nm, em slit 10 nm, PMT 1000 V; Sunset Yellow FCF: excitation 482 nm, ex/em slits 10 nm, PMT 930V; Chocolate Brown: excitation 463 nm, ex slit 5 nm, em slit 10 nm, PMT 1000 V; Azorubine: excitation 512 nm, ex slit 5 nm, em slit 10 nm, PMT 1000 V; Amaranth: excitation 522 nm, ex slit 10 nm, em slit 10 nm, PMT 1000 V; Ponceau 4R: excitation 513 nm, ex slit 10 nm, em slit 10 nm, PMT 950 V; Brilliant Blue FCF: excitation 628 nm, ex slit 5 nm, em slit 10 nm, PMT 970 V; Bromophenol Blue: excitation 590 nm, ex slit 5 nm, em slit 5 nm, PMT 1000 V; Chlorophenol Red: excitation 575 nm, ex slit 5 nm, em slit 5 nm, PMT 1000 V; Coomassie G‐250: excitation 580 nm, ex slit 5 nm, em slit 5 nm, PMT 1000 V; DFHBI‐1T: excitation 420 nm, ex slit 5 nm, em slit 5 nm, PMT 1000 V; and Xylene Cyanole FF: excitation 614 nm, ex slit 5 nm, em slit 5 nm, PMT 1000 V.

#### 4.1.18 Liposome Microscopy

Liposomes encapsulating transcription reactions with 130 nM dsDNA template for either Honeydew or R8‐04 were prepared using 2.739 mM DOPC, 2.739 mM POPC, 3.3 mM POPE, 2.2 mM POPG, 2 µM PEG350 PE, and 20 µM Alexa Fluor 594 PE with 3.3 mM cholesterol as described previously [66]. Briefly, the lipid–oil solution was layered on top of a volume of isotonic exogenous buffer in a collection tube, above which a capillary device was loaded with transcription reaction mix amended with 50 µM poloxamer 188. The exogenous medium contained a working concentration of 50 µM poloxamer 188 and was composed and adjusted to a final isotonic osmolarity of approximately 815 mOsmol with a trim solution of HEPES (pH 7.9) and glucose in a 1:18 molar ratio. To form the liposomes, the loaded spin column device was centrifuged at 31 RCF for 10 min with a rotor temperature of 25 °C; the aqueous bottom layer containing formed liposomes was collected after an additional postformation pelleting spin at 300 RCF for 5 min before incubation at 37 °C for 3 h to initiate transcription within the liposomes. Microscope slides were prepared by rinsing with acetone, 70% EtOH, and drying, prior to liposome mounting in a frame seal chamber. Samples were imaged on a Nikon Eclipse Ti‐E microscope equipped with a Hamamatsu Flash v3 camera (Hamamatsu) and a Sola light engine (Lumencor) was used as an epifluorescent light source. An appropriate filter cube was used to visualize the fluorescence of the Alexa Fluor 594 labeled liposome membranes. The 440 nm channel of the light engine at 40% intensity was used to excite the tartrazine aptamer, a filter cube for the FITC channel was modified by substituting the excitation filter with a 500 nm shortpass filter (FESH0500, Thorlabs), and epifluorescence was observed with a consistent exposure time of 100 ms and 16‐bit 2 × 2 binning.

Multichannel micrographs were exported as ND2 files and then each channel was exported as a tiff file for processing with FIJI. Three custom FIJI macros were written to measure the lumen signal of the encapsulated transcription reaction. The membrane channel was processed to produce a binary mask that is used to define regions of interest in the lumen channel micrographs. Integrated density of the liposome lumens was measured using the mask‐identified membrane regions of interest secondary to denoising using the PureDenoise plugin.

## Funding

This study was supported by National Institutes of Health (GM152459) and ARPA‐H (B674286).

## Conflicts of Interest

The authors declare no conflicts of interest.

## Supporting information

Supplementary Material

## Data Availability

The data that supports the findings of this study are available in the Supporting Information of this article. DNA sequencing reads of selection rounds have been deposited in the Sequencing Read Archive (SRA) at accession PRJNA1490713.

## References

[1] E. Bertrand , P. Chartrand , M. Schaefer , S. M. Shenoy , R. H. Singer , and R. M. Long , “Localization of ASH1 mRNA Particles in Living Yeast,” Molecular Cell 2 (1998): 437–445. 9809065 10.1016/s1097-2765(00)80143-4

[2] D. Fusco , N. Accornero , B. Lavoie , et al., “Single mRNA Molecules Demonstrate Probabilistic Movement in Living Mammalian Cells,” Current Biology 13 (2003): 161–167. 12546792 10.1016/s0960-9822(02)01436-7 PMC4764064

[3] T. Lionnet , K. Czaplinski , X. Darzacq , et al., “A Transgenic Mouse for In Vivo Detection of Endogenous Labeled mRNA,” Nature Methods 8 (2011): 165–170. 21240280 10.1038/nmeth.1551 PMC3076588

[4] K.‐R. Luo , N.‐C. Huang , and T.‐S. Yu , “Selective Targeting of Mobile mRNAs to Plasmodesmata for Cell‐to‐Cell Movement,” Plant Physiology 177 (2018): 604–614. 29581179 10.1104/pp.18.00107 PMC6001314

[5] J. F. Garcia and R. Parker , “Ubiquitous Accumulation of 3′ mRNA Decay Fragments in *Saccharomyces Cerevisiae* mRNAs with Chromosomally Integrated MS2 Arrays,” RNA 22 (2016): 657–659. 27090788 10.1261/rna.056325.116 PMC4836640

[6] G. Haimovich , D. Zabezhinsky , B. Haas , et al., “Use of the MS2 Aptamer and Coat Protein for RNA Localization in Yeast: A Response to “MS2 Coat Proteins Bound to Yeast mRNAs Block 5′ to 3′ Degradation and Trap mRNA Decay Products: Implications for the Localization of mRNAs by MS2‐MCP System”,” RNA 22 (2016): 660–666. 26968626 10.1261/rna.055095.115 PMC4836641

[7] D. A. Zacharias , J. D. Violin , A. C. Newton , and R. Y. Tsien , “Partitioning of Lipid‐Modified Monomeric GFPs into Membrane Microdomains of Live Cells,” Science 296 (2002): 913–916. 11988576 10.1126/science.1068539

[8] E. L. Snapp , R. S. Hegde , M. Francolini , et al., “Formation of Stacked ER Cisternae by Low Affinity Protein Interactions,” The Journal of Cell Biology 163 (2003): 257–269. 14581454 10.1083/jcb.200306020 PMC2173526

[9] K. D. Warner , M. C. Chen , W. Song , et al., “Structural Basis for Activity of Highly Efficient RNA Mimics of Green Fluorescent Protein,” Nature Structural & Molecular Biology 21 (2014): 658–663. 10.1038/nsmb.2865 PMC4143336 25026079

[10] H. Huang , N. B. Suslov , N.‐S. Li , et al., “A G‐Quadruplex–containing RNA Activates Fluorescence in a GFP‐Like Fluorophore,” Nature Chemical Biology 10 (2014): 686–691. 24952597 10.1038/nchembio.1561 PMC4104137

[11] K. Huang , X. Chen , C. Li , et al., “Structure‐Based Investigation of Fluorogenic Pepper Aptamer,” Nature Chemical Biology 17 (2021): 1289–1295. 34725509 10.1038/s41589-021-00884-6

[12] C. Baugh , D. Grate , and C. Wilson , “2.8 Å Crystal Structure of the Malachite Green Aptamer 1 1Edited by J. A. Doudna,” Journal of Molecular Biology 301 (2000): 117–128. 10926496 10.1006/jmbi.2000.3951

[13] K. D. Warner , L. Sjekloća , W. Song , G. S. Filonov , S. R. Jaffrey , and A. R. Ferré‐D’Amaré , “A Homodimer Interface without Base Pairs in an RNA Mimic of Red Fluorescent Protein,” Nature Chemical Biology 13 (2017): 1195–1201. 28945234 10.1038/nchembio.2475 PMC5663454

[14] R. J. Trachman 3rd , R., Cojocaru , D., Wu , et al., “Structure‐Guided Engineering of the Homodimeric Mango‐IV Fluorescence Turn‐on Aptamer Yields an RNA FRET Pair,” Structure 28 (2020): 776–785.e3. 32386573 10.1016/j.str.2020.04.007 PMC7347457

[15] R. J., Trachman 3rd , N. A., Demeshkina , M. W. L., Lau , et al., “Structural Basis for High‐Affinity Fluorophore Binding and Activation by RNA Mango,” Nature Chemical Biology 13 (2017): 807–813. 28553947 10.1038/nchembio.2392 PMC5550021

[16] R. J. Trachman and A. R. Ferré‐D’Amaré , “Tracking RNA with Light: Selection, Structure, and Design of Fluorescence Turn‐on RNA Aptamers,” Quarterly Reviews of Biophysics 52 (2019):e8. 31423956 10.1017/S0033583519000064 PMC7377909

[17] G. Aquino‐Jarquin and J. D. Toscano‐Garibay , “RNA Aptamer Evolution: Two Decades of SELEction,” International Journal of Molecular Sciences 12 (2011): 9155–9171. 22272125 10.3390/ijms12129155 PMC3257122

[18] E. Duchardt‐Ferner , M. Juen , B. Bourgeois , et al., “Structure of an RNA Aptamer in Complex with the Fluorophore Tetramethylrhodamine,” Nucleic Acids Research 48 (2020): 949–961. 31754719 10.1093/nar/gkz1113 PMC6954400

[19] K. L. Litvinenko , N. M. Webber , and S. R. Meech , “Internal Conversion in the Chromophore of the Green Fluorescent Protein: Temperature Dependence and Isoviscosity Analysis,” The Journal of Physical Chemistry A 107 (2003): 2616–2623.

[20] T. P. Constantin , G. L. Silva , K. L. Robertson , et al., “Synthesis of New Fluorogenic Cyanine Dyes and Incorporation into RNA Fluoromodules,” Organic Letters 10 (2008): 1561–1564. 18338898 10.1021/ol702920e

[21] J. R. Babendure , S. R. Adams , and R. Y. Tsien , “Aptamers Switch on Fluorescence of Triphenylmethane Dyes,” Journal of the American Chemical Society 125 (2003): 14716–14717. 14640641 10.1021/ja037994o

[22] Quest GraphTM Spectrum [Malachite green], 2026.

[23] T. Wang , PhD Thesis (Iowa State University, 2008).

[24] W. Song , R. L. Strack , N. Svensen , and S. R. Jaffrey , “Plug‐and‐Play Fluorophores Extend the Spectral Properties of Spinach,” Journal of the American Chemical Society 136 (2014): 1198–1201. 24393009 10.1021/ja410819x PMC3929357

[25] G. S. Filonov , J. D. Moon , N. Svensen , and S. R. Jaffrey , “Broccoli: Rapid Selection of an RNA Mimic of Green Fluorescent Protein by Fluorescence‐Based Selection and Directed Evolution,” Journal of the American Chemical Society 136 (2014): 16299–16308. 25337688 10.1021/ja508478x PMC4244833

[26] Z. Chen , W. Chen , Z. Reheman , H. Jiang , J. Wu , and X. Li , “Genetically Encoded RNA‐Based Sensors with Pepper Fluorogenic Aptamer,” Nucleic Acids Research 51 (2023): 8322–8336. 37486780 10.1093/nar/gkad620 PMC10484673

[27] E. V. Dolgosheina , S. C. Y. Jeng , S. S. S. Panchapakesan , et al., “RNA Mango Aptamer‐Fluorophore: A Bright, High‐Affinity Complex for RNA Labeling and Tracking,” ACS Chemical Biology 9 (2014): 2412–2420. 25101481 10.1021/cb500499x

[28] A. Autour , S. C. Y. Jeng , A. D. Cawte , et al., “Fluorogenic RNA Mango Aptamers for Imaging Small Non‐Coding RNAs in Mammalian Cells,” Nature Communications 9 (2018): 1–12. 10.1038/s41467-018-02993-8 PMC5811451 29440634

[29] C. Steinmetzger , I. Bessi , A.‐K. Lenz , and C. Höbartner , “Structure–fluorescence Activation Relationships of a Large Stokes Shift Fluorogenic RNA Aptamer,” Nucleic Acids Research 47, no. 22 (2019): 11538–11550. 31740962 10.1093/nar/gkz1084 PMC7145527

[30] Z. Chen , W. Chen , C. Xu , et al., “Near‐Infrared Fluorogenic RNA for In Vivo Imaging and Sensing,” Nature Communications 16 (2025): 518. 10.1038/s41467-024-55093-1 PMC11718054 39788937

[31] J. B. Segur and H. E. Oberstar , “Viscosity of Glycerol and Its Aqueous Solutions,” Industrial and Engineering Chemistry 43 (1951): 2117–2120.

[32] X. Chen , D. Zhang , N. Su , et al., “Visualizing RNA Dynamics in Live Cells with Bright and Stable Fluorescent RNAs,” Nature Biotechnology 37 (2019): 1287–1293. 10.1038/s41587-019-0249-1 31548726

[33] L. Jiang , X. Xie , N. Su , et al., “Large Stokes Shift Fluorescent RNAs for Dual‐Emission Fluorescence and Bioluminescence Imaging in Live Cells,” Nature Methods 20 (2023): 1563–1572. 37723244 10.1038/s41592-023-01997-7

[34] M. Sunbul and A. Jäschke , “SRB‐2: A Promiscuous Rainbow Aptamer for Live‐Cell RNA Imaging,” Nucleic Acids Research 46 (2018): e110. 29931157 10.1093/nar/gky543 PMC6182184

[35] D. Grate and C. Wilson , “Laser‐Mediated, Site‐Specific Inactivation of RNA Transcripts,” Proceedings of the National Academy of Sciences of the United States of America 96 (1999): 6131–6136. 10339553 10.1073/pnas.96.11.6131 PMC26847

[36] A. O. de Almada Vilhena , K. M. M. Lima , L. F. C. de Azevedo , et al., “The Synthetic Dye Malachite Green Found in Food Induces Cytotoxicity and Genotoxicity in Four Different Mammalian Cell Lines from Distinct Tissuesw,” Toxicology Research 12 (2023): 693–701. 37663817 10.1093/toxres/tfad059 PMC10470350

[37] N. J. Gaut , J. Gomez‐Garcia , J. M. Heili , et al., “Programmable Fusion and Differentiation of Synthetic Minimal Cells,” ACS Synthetic Biology 11 (2022): 855–866. 35089706 10.1021/acssynbio.1c00519

[38] J. M. Heili , K. Stokes , N. J. Gaut , et al., “Controlled Exchange of Protein and Nucleic Acid Signals from and between Synthetic Minimal Cells,” Cell Systems 15 (2024): 49–62.e4. 38237551 10.1016/j.cels.2023.12.008

[39] W. Song , G. S. Filonov , H. Kim , et al., “Imaging RNA Polymerase III Transcription Using a Photostable RNA–fluorophore Complex,” Nature Chemical Biology 13 (2017): 1187–1194. 28945233 10.1038/nchembio.2477 PMC5679246

[40] J. S. Paige , K. Y. Wu , and S. R. Jaffrey , “RNA Mimics of Green Fluorescent Protein,” Science 333 (2011): 642–646. 21798953 10.1126/science.1207339 PMC3314379

[41] S. K. Dey , G. S. Filonov , A. O. Olarerin‐George , B. T. Jackson , L. W. S. Finley , and S. R. Jaffrey , “Repurposing an Adenine Riboswitch into a Fluorogenic Imaging and Sensing Tag,” Nature Chemical Biology 18 (2022): 180–190. 34937909 10.1038/s41589-021-00925-0 PMC8967656

[42] T. S. Lotz , T. Halbritter , C. Kaiser , et al., “A Light‐Responsive RNA Aptamer for an Azobenzene Derivative,” Nucleic Acids Research 47 (2019): 2029–2040. 30517682 10.1093/nar/gky1225 PMC6393235

[43] S. R. Shafqat , S. A. Bhawani , S. Bakhtiar , and M. N. M. Ibrahim , “Synthesis of Molecularly Imprinted Polymer for Removal of Congo Red,” BMC Chemistry 14 (2020): 27. 32266334 10.1186/s13065-020-00680-8 PMC7118869

[44] S. R. Shafqat , S. A. Bhawani , S. Bakhtiar , M. N. M. Ibrahim , and S. S. Shafqat , “Template‐Assisted Synthesis of Molecularly Imprinted Polymers for the Removal of Methyl Red from Aqueous Media,” BMC Chemistry 17 (2023): 46. 37165372 10.1186/s13065-023-00957-8 PMC10173658

[45] A. B. Kinghorn , W. Guo , L. Wang , et al., “Evolution Driven Microscale Combinatorial Chemistry in Intracellular Mimicking Droplets to Engineer Thermostable RNA for Cellular Imaging,” Small 21 (2025): e2409911. 39865936 10.1002/smll.202409911 PMC11878259

[46] R. Cubi , F. Bouhedda , M. Collot , A. S. Klymchenko , and M. Ryckelynck , “µIVC‐Useq: A Microfluidic‐Assisted High‐Throughput Functional Screening in Tandem with Next‐Generation Sequencing and Artificial Neural Network to Rapidly Characterize RNA Molecules,” RNA 27 (2021): 841–853. 33952671 10.1261/rna.077586.120 PMC8208054

[47] A. R. Paul , M. Falsaperna , H. Lavender , M. D. Garrett , and C. J. Serpell , “Selection of Optimised Ligands by Fluorescence‐Activated Bead Sorting,” Chemical Science 14 (2023): 9517–9525. 37712023 10.1039/d3sc03581f PMC10498682

[48] M. Gotrik , G. Sekhon , S. Saurabh , M. Nakamoto , M. Eisenstein , and H. T. Soh , “Direct Selection of Fluorescence‐Enhancing RNA Aptamers,” Journal of the American Chemical Society 140 (2018): 3583–3591. 29505267 10.1021/jacs.7b10724

[49] R. J., Trachman 3rd , A., Autour , S. C. Y., Jeng , et al., “Structure and Functional Reselection of the Mango‐III Fluorogenic RNA Aptamer,” Nature Chemical Biology 15 (2019): 472–479. 30992561 10.1038/s41589-019-0267-9 PMC7380332

[50] H. Kim and S. R. Jaffrey , “A Fluorogenic RNA‐Based Sensor Activated by Metabolite‐Induced RNA Dimerization,” Cell Chemical Biology 26 (2019): 1725–1731.e6. 31631009 10.1016/j.chembiol.2019.09.013 PMC6939632

[51] L. Truong , H. Kooshapur , S. K. Dey , et al., “The Fluorescent Aptamer Squash Extensively Repurposes the Adenine Riboswitch Fold,” Nature Chemical Biology 18 (2022): 191–198. 34937911 10.1038/s41589-021-00931-2 PMC9812287

[52] C. Steinmetzger , N. Palanisamy , K. R. Gore , and C. Höbartner , “A Multicolor Large Stokes Shift Fluorogen‐Activating RNA Aptamer with Cationic Chromophores,” Chemistry – A European Journal 25 (2019): 1931–1935. 30485561 10.1002/chem.201805882

[53] J. M. Heili , J. Gomez‐Garcia , N. J. Gaut , et al., “Real‐Time Visualization of *In* *Vitro* Transcription of a Fluorescent RNA Aptamer: An Experiment for the Upper‐Division Undergraduate or First‐Year Graduate Laboratory,” Journal of Chemical Education 95 (2018): 1867–1871.

[54] J. M. Heili , K. P. Adamala , and A. E. Engelhart , “Activation of Caged Functional RNAs by An Oxidative Transformation,” ChemBioChem 26 (2025): e202401056. 39740778 10.1002/cbic.202401056 PMC12007075

[55] J. H. Davis and J. W. Szostak , “Isolation of High‐Affinity GTP Aptamers from Partially Structured RNA Libraries,” Proceedings of the National Academy of Sciences of the United States of America 99 (2002): 11616–11621. 12185247 10.1073/pnas.182095699 PMC129318

[56] M. Martin , “Cutadapt Removes Adapter Sequences from High‐Throughput Sequencing Reads,” EMBnet Journal 17 (2011): 10.

[57] G. J. Hannon , FastX‐Toolkit, http://hannonlab.cshl.edu/fastx_toolkit, n.d.

[58] K. K. Alam , J. L. Chang , and D. H. Burke , “FASTAptamer: A Bioinformatic Toolkit for High‐Throughput Sequence Analysis of Combinatorial Selections,” Molecular Therapy ‐ Nucleic Acids 4 (2015): e230. 25734917 10.1038/mtna.2015.4 PMC4354339

[59] S. T. Kramer , P. R. Gruenke , K. K. Alam , D. Xu , and D. H. Burke , “FASTAptameR 2.0: A Web Tool for Combinatorial Sequence Selections,” Molecular Therapy ‐ Nucleic Acids 29 (2022): 862–870. 36159593 10.1016/j.omtn.2022.08.030 PMC9464650

[60] T. L. Bailey , J. Johnson , C. E. Grant , and W. S. Noble , “The MEME Suite,” Nucleic Acids Research 43 (2015): W39–W49. 25953851 10.1093/nar/gkv416 PMC4489269

[61] T. L. Bailey , “STREME: Accurate and Versatile Sequence Motif Discovery,” Bioinformatics 37 (2021): 2834–2840. 33760053 10.1093/bioinformatics/btab203 PMC8479671

[62] R. Lorenz , S. H. Bernhart , C. H. Z. Siederdissen , et al., “ViennaRNA Package 2.0,” Algorithms for Molecular Biology 6 (2011): 26. 22115189 10.1186/1748-7188-6-26 PMC3319429

[63] I. L. Hofacker , W. Fontana , P. F. Stadler , L. S. Bonhoeffer , M. Tacker , and P. Schuster , “Fast Folding and Comparison of RNA Secondary Structures,” Monatshefte Fur Chemie 125 (1994): 167–188.

[64] X. Qu and J. B. Chaires , “Methods in Enzymology,” Methods in Enzymology 321 (2000): 353–369. 10909066 10.1016/s0076-6879(00)21202-0

[65] G. Duportail , Y. Mauss , and J. Chambron , “Quantum Yields and Fluorescence Lifetimes of Acridine Derivatives Interacting with DNA,” Biopolymers 16 (1977): 1397–1413. 880364 10.1002/bip.1977.360160703

[66] O. M. Venero , W. Sato , J. M. Heili , C. Deich , and K. P. Adamala , “Methods in Molecular Biology,” Methods in Molecular Biology 2433 (2022): 227–235. 34985748 10.1007/978-1-0716-1998-8_14

