# Supplementary material for: Honeydew: A Fluorescent Aptamer for Low‐Cost Azo Food Dyes
Source: Chembiochem. 2026 Jul 26;27(14):e70471. doi: 10.1002/cbic.70471 (PMC13401960; doi:10.1002/cbic.70471)
Supplement: Supplementary file 1 — Supplementary Material [file CBIC-27-e70471-s001.pdf]

Supporting Information
for
# A fluorescent aptamer for low-cost azo food dyes

Joseph M. Heili[1], Paul Mroch[1], Tanner G. Hoog[1], Orion M. Venero[1], Grace M. Gustafson[1], Katarzyna P. Adamala[1,2], Aaron E. Engelhart[1,2]*

[1]Department of Genetics, Cell Biology, and Development, University of Minnesota, Minneapolis, MN 55455, USA. [2]Department of Biochemistry, Molecular Biology and Biophysics, University of Minnesota, Minneapolis, Minnesota 55455, USA [*]To whom correspondence should be addressed: enge0213@umn.edu

# Table of Contents


**Supplementary Figure 1.** ~~Sequences screened~~Viscosity and solvent effects on tartrazine fluorescence~~ screening results~~.

**Supplementary Figure 2.** Sequences screened and fluorescence screening results.

**Supplementary Figure 3.** Transcription purification gel and yield for Honeydew and R8-04

**Supplementary Figure ~~3.~~4.** Fluorescence of truncated Honeydew sequences.

**Supplementary Figure 5.** Kinetic monitoring of tartrazine fluorescence activation by Honeydew.

**Supplementary Figure 6.** Honeydew-azo dye fluorescence photography, absorbance and fluorescence spectra

**Supplementary Figure ~~4~~7.** Fluorescence ~~microscopy~~enhancement of various dyes by Honeydew ~~transcription liposomes, AF594 channel~~aptamer

**Supplementary Figure ~~5~~8.** Fluorescence microscopy of Honeydew transcription liposomes, AF594 channel

**Supplementary Figure 9.** Fluorescence microscopy of R8-04 transcription liposomes, AF594 channel

**Table 1.** Oligonucleotides used

**Supplementary Methods**

Viscosity and solvent effects on tartrazine

Sequencing analysis criteria

HoneydewCountCompiler.py script

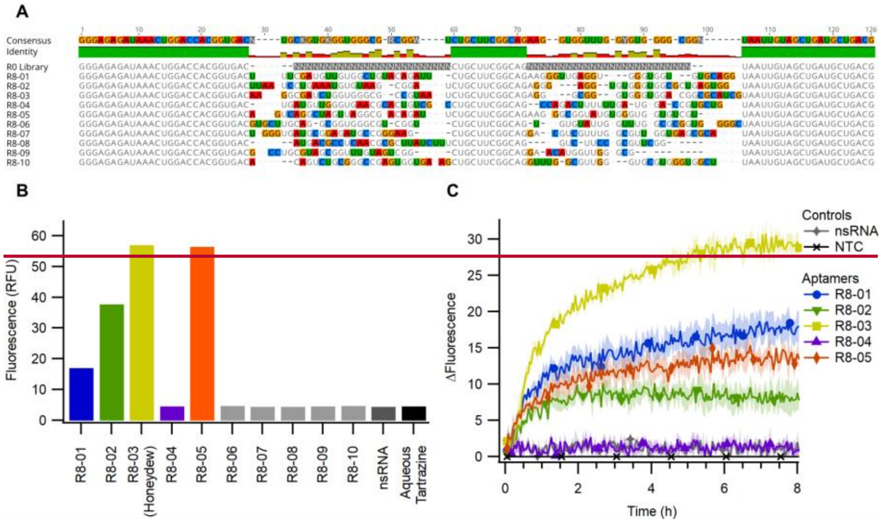


~~Supplementary Figure 1~~Honeydew truncation sequence transcription reactions

Fluorescence binding kinetics

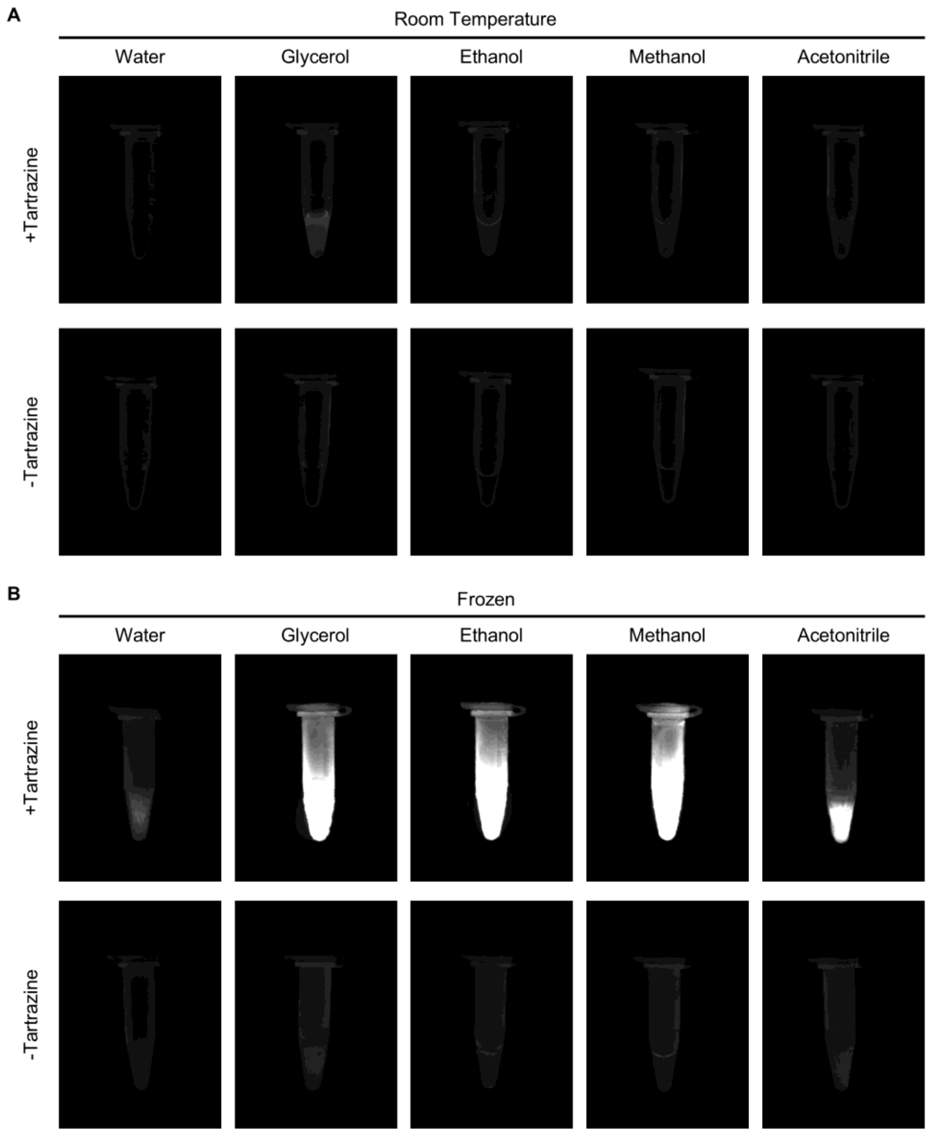


**Supplementary Figure 1: Viscosity and solvent effects on tartrazine fluorescence.** Fluorescence imaging of 10 μM tartrazine (top rows) or no-dye controls (bottom rows) in water, glycerol, ethanol, methanol, and acetonitrile. **(A)** Samples imaged at room temperature. All images

in panel collected and processed at identical conditions and settings. **(B)** Samples imaged while frozen via liquid nitrogen. All images in panel collected and processed at identical conditions and settings.

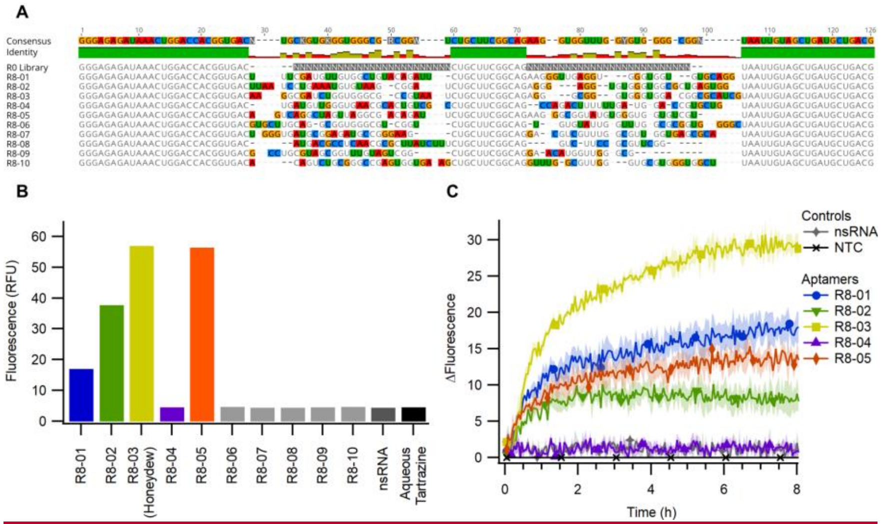


**Supplementary Figure 2: Sequences screened and fluorescence screening results. (A)** Sequences of all initial aptamer candidates identified via sequencing analysis, both for RPM/enrichment based criteria and motif search criteria, after eight rounds of selection. Alignment via Geneious version 10.2. **(B)** Fluorescence (excitation 420 nm, emission recorded at 550 nm) of the ten initial round 8 aptamer candidate RNA sequences (50 μM) in selection buffer with tartrazine (50 μM). Sequences R8-01 through -05 in saturated colors were chosen for characterization based on RPM and round-by-round enrichment, while sequences R8-06 through R8-10 in light gray were chosen based on clustering analysis and motif search. An RNA sequence that was not present in the selection library is shown in gray. Aqueous tartrazine in

selection buffer with no RNA present is shown in black. **(C)** Fluorescence monitored transcription reactions in the presence of tartrazine (100 μM) for the non-selection-RNA (gray diamonds), no template control (black ~~x's~~crosses), and five aptamer candidates chosen for characterization based on RPM and enrichment. Fluorescence was detected on a plate reader with excitation at 420 nm and emission recorded at 550 nm. Fluorescence values for the NTC were used as a baseline. The shaded area represents S.E.M., n=3.

A

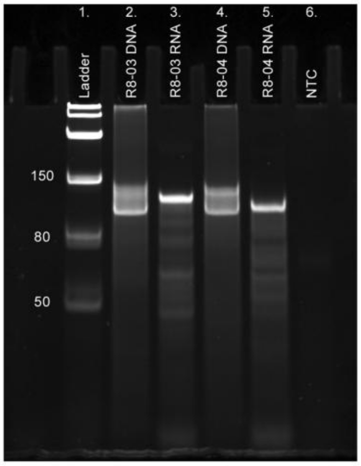


B

| Gel Lane | Sample Name | Purification Yield (µg) |
|---|---|---|
| 1 | ssRNA Ladder | n/a |
| 2 | R8-03 DNA | n/a |
| 3 | R8-03 RNA (Honeydew) | 561 |
| 4 | R8-04 DNA | n/a |
| 5 | R8-04 RNA | 624 |
| 6 | NTC | 15 |

**Supplementary Figure ~~2~~3: Transcription purification gel and yield for Honeydew and R8-04. (A)** Denaturing Urea-PAGE showing 100 ng samples of R8-03 (Honeydew) dsDNA transcription template, R8-03 purified transcription RNA product, R8-04 dsDNA transcription template, R8-04 purified transcription RNA product, and purified control transcription containing no template dsDNA (NTC). **(B)** Table showing DNA/RNA oligos loaded into each gel lane and total RNA purification yields from the applicable transcriptions.

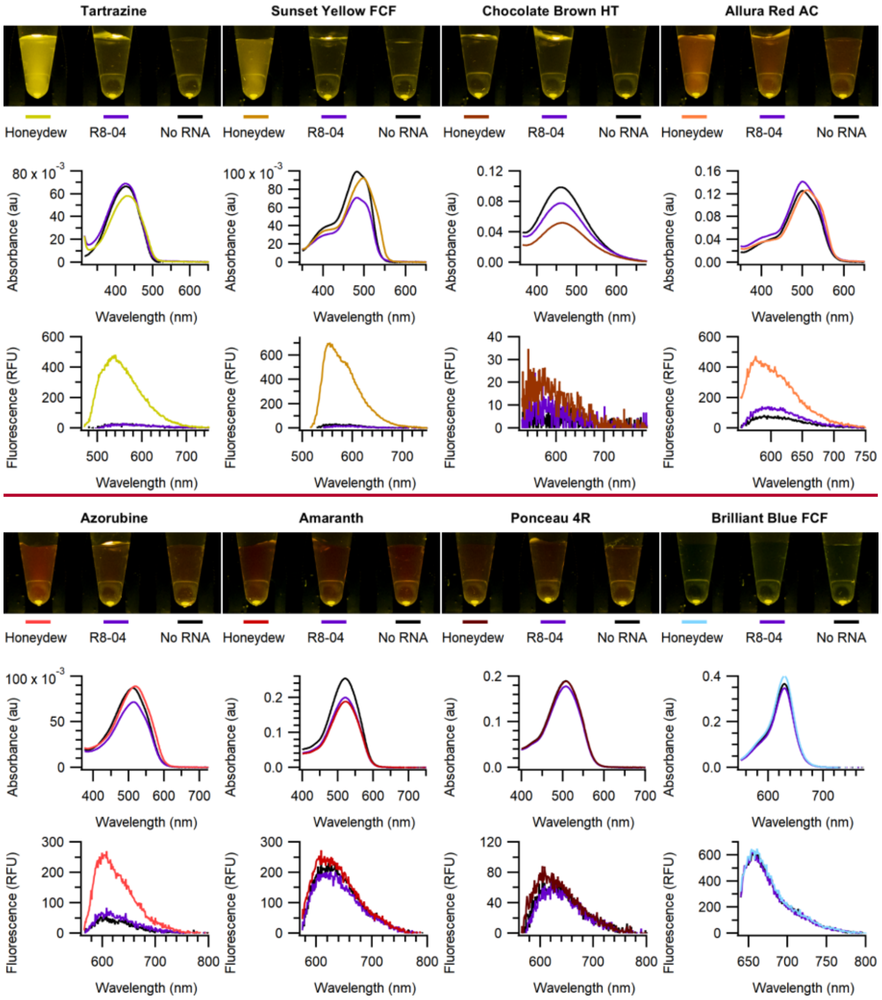
Tartrazine
Sunset Yellow FCF
Chocolate Brown HT
Allura Red AC
Honeydew
R8-04
No RNA
Absorbance (au)
Fluorescence (RFU)
Wavelength (nm)
Azorubine
Amaranth
Ponceau 4R
Brilliant Blue FCF

Supplementary Figure 3

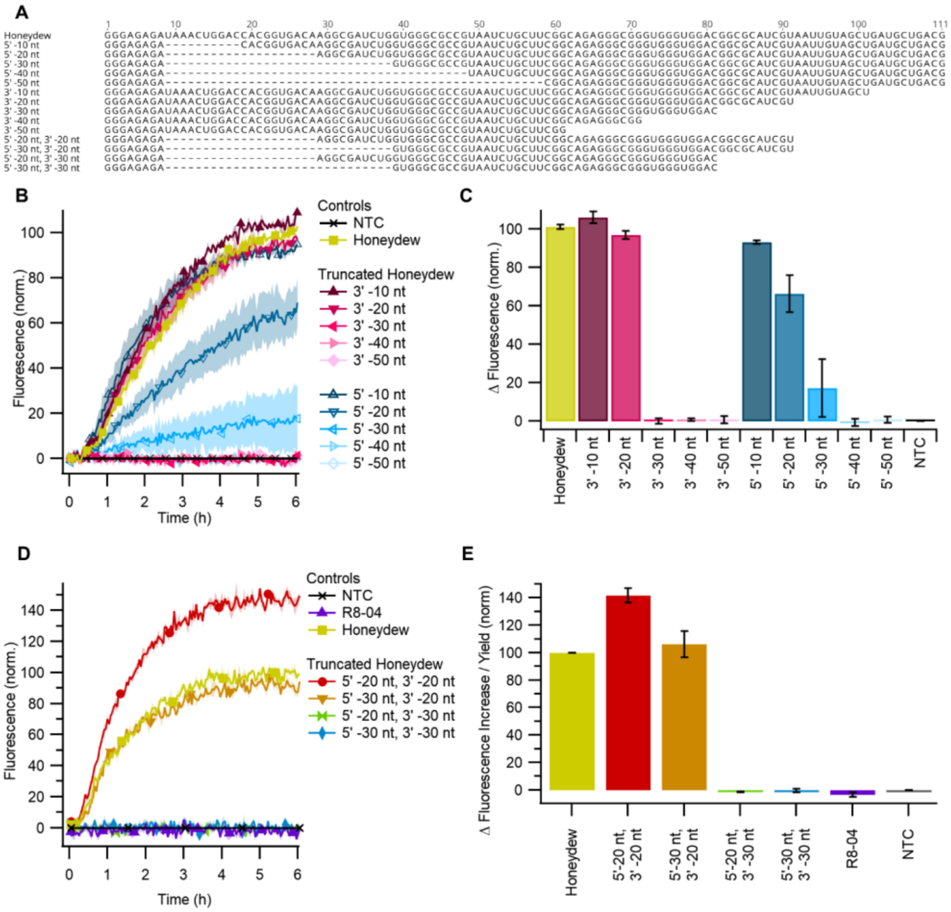


**Supplementary Figure 4: Fluorescence of truncated Honeydew sequences. (A)** Alignment of truncated Honeydew sequences screened for activation of tartrazine fluorescence. **(B)** Real-time fluorescence monitoring of *in vitro* transcription reactions producing single-end Honeydew truncation sequences in the presence of tartrazine (100 μM). Truncations were carried out in ten nucleotide increments from either the 5′ or 3′ end of the sequence. Fluorescence was detected on

a plate reader with excitation at 420 nm and emission recorded at 550 nm. Fluorescence values were normalized to the NTC and full length Honeydew sequence. Shaded areas represents S.E.M., n=3. **(C)** Endpoint fluorescence for the transcription reactions shown in (B) normalized to the signals of the NTC and full length Honeydew sequence. Bars represent S.E.M calculated with n=3. **(D)** Fluorescence monitored transcription reactions in the presence of tartrazine (100 µM) of 5′/3′ double truncation Honeydew sequences based on the results of single truncation sequences from (A). Fluorescence was detected on a plate reader with excitation at 420 nm and emission recorded at 550 nm. Fluorescence values were normalized to the NTC and full length Honeydew sequence. Shaded areas represent S.E.M., n=3. **(E)** Endpoint fluorescence for the transcription reactions shown in (D) and analyzed as a ratio of the purification yield post transcription to account for differences in fluorescence due to variations in sequence transcription rates. Normalized to the signals of the NTC and full length Honeydew sequence. Bars represent S.E.M calculated with n=3.

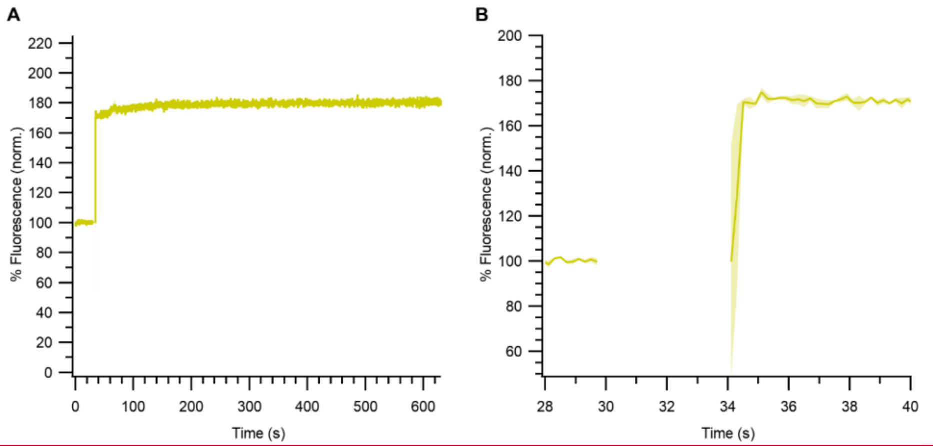


**Supplementary Figure 5: Kinetic monitoring of tartrazine fluorescence activation by Honeydew. (A)** Real-time fluorescence of 2.5 µM tartrazine monitored over ten minutes. The cuvette was briefly removed from the optical path at 30 seconds prior to mix in Honeydew aptamer. Data were normalized to the pre-mixing fluorescence values. Shaded area represents S.E.M., n = 3. **(B)** Zoomed-in view of the mixing period, demonstrating that ~100% of fluorescence enhancement occurred within the ~4 second mixing period.

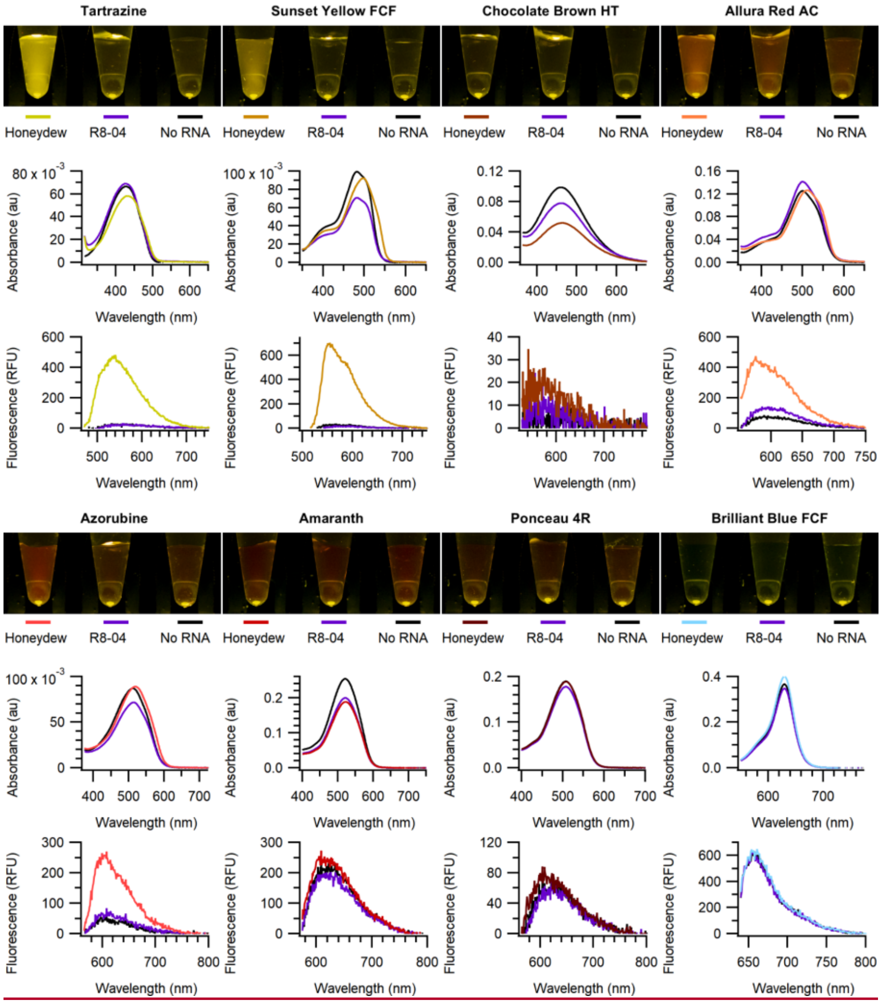


**Supplementary Figure 6: Honeydew-azo dye fluorescence photography, absorbance and fluorescence spectra.** Fluorescence photography and absorbance/fluorescence spectroscopy of Honeydew (20 µM) or R8-04 (20 µM) in the presence of azo food dyes (10 µM). Fluorescence photography of sets of three tubes per specified azo dyes, with Honeydew (left tube), R8-04 (middle tube), and no added RNA (right tube) in the presence of each dye. Illuminated under

identical conditions with a blue light gel transilluminator and photographed using an orange emission filter. All images were captured and processed using identical settings. Under each set of tubes is the corresponding absorbance and fluorescence spectra for the samples photographed.

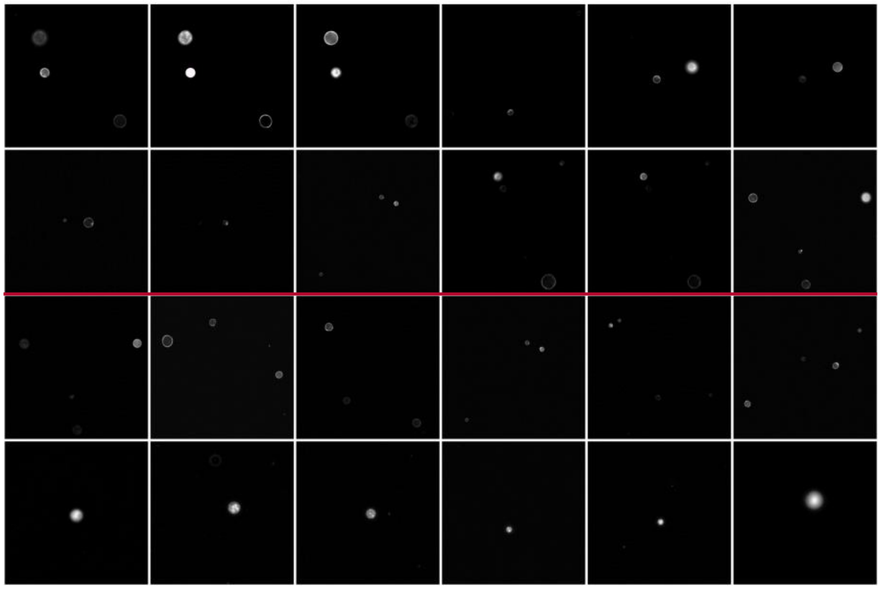

Supplementary Figure 4

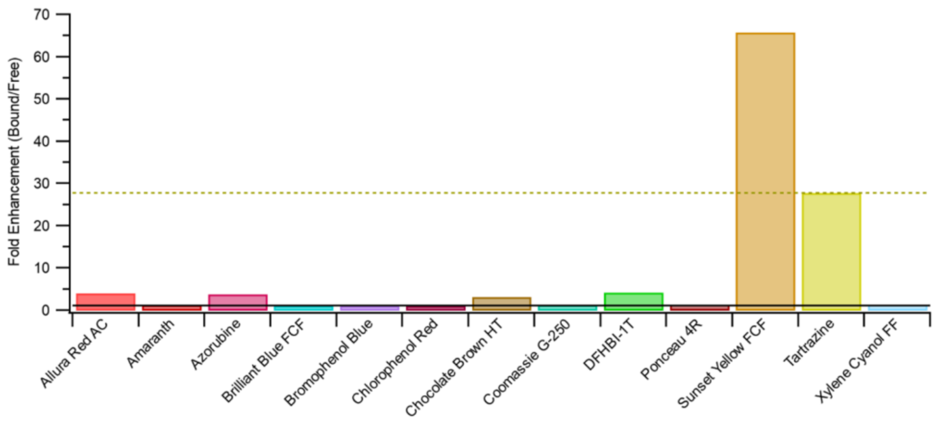


**Supplementary Figure 7: Fluorescence enhancement of various dyes by Honeydew aptamer.** Fold activation of fluorescence for 10 µM dye in the presence of 20 µM Honeydew, calculated relative to the baseline fluorescence of each free dye. The black horizontal line denotes a fold enhancement of 1 (no enhancement), while the yellow dotted horizontal line denotes the enhancement level of Honeydew-tartrazine.

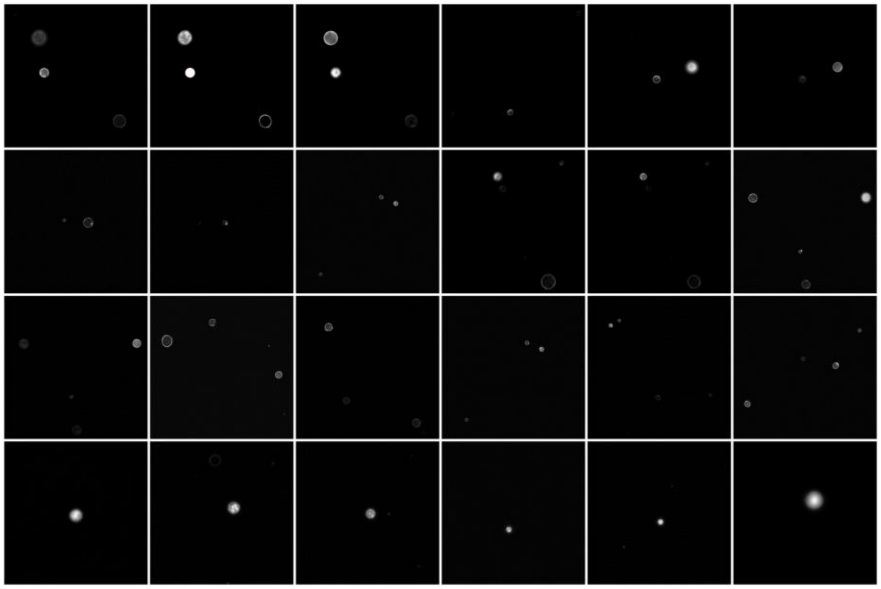

**Supplementary Figure 8: Fluorescence microscopy of Honeydew transcription liposomes, AF594 channel**

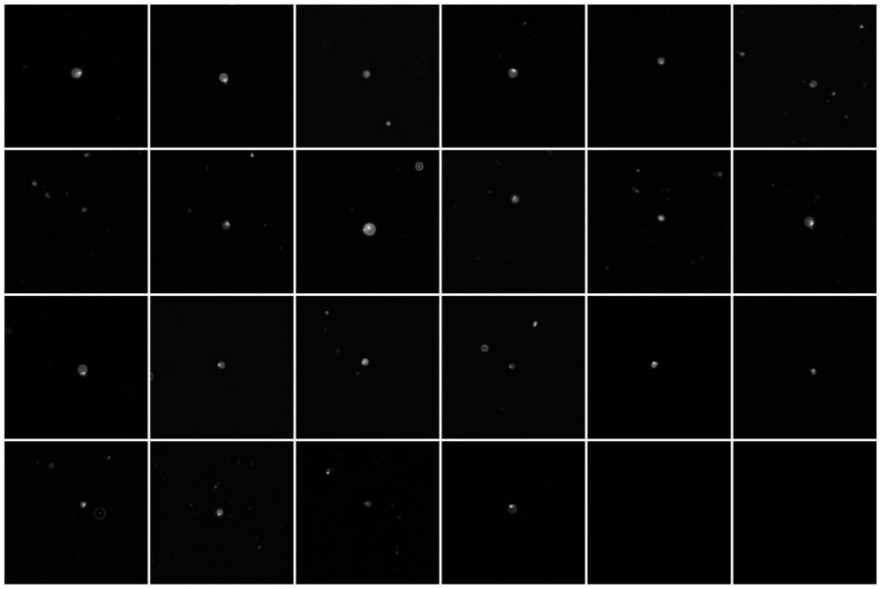

**Supplementary Figure ~~5~~9: Fluorescence microscopy of R8-04 transcription liposomes, AF594 channel**

**Formatted:** Font: Bold

**Formatted:** Left, Indent: First line: 0", Space After: 8 pt, Line spacing: Multiple 1.16 li

| ~~Oligo~~ Name | Sequence (~~5' -> 3')~~5′ -> 3′) |
|---|---|
| R0-Library Template | GGG AGA GAT AAA CTG GAC CAC GGT GAC NNN NNN NNN NNN NNN NNN NNN NNN NCT GCT TCG GCA GNN NNN NNN NNN NNN NNN NNN NNN NNN TAA TTG TAG CTG ATG CTG ACG |
| R8-01 | GGG AGA GAU AAA CUG GAC CAC GGU GAC UUU CGA UGU UGU GGC UGU ACA GAU UCU GCU UCG GCA GAA GGG UUG AGG UGG GUG GUG UGC AGG UAA UUG UAG CUG AUG CUG ACG |
| R8-02 | GGG AGA GAU AAA CUG GAC CAC GGU GAC UUA AUC CUG AAA UGU GUA AGC GGA UCU GCU UCG GCA GAG GGA GGU GUG GGU GGC GCU GAG UGG UAA UUG UAG CUG AUG CUG ACG |
| R8-03 (Honeydew) | GGG AGA GAU AAA CUG GAC CAC GGU GAC AAG GCG AUC UGG UGG GCG CCG UAA UCU GCU UCG GCA GAG GGC GGG UGG GUG GAC GGC GCA UCG UAA UUG UAG CUG AUG CUG ACG |
| Honeydew 5′ -10 nt | GGG AGA GAC ACG GUG ACA AGG CGA UCU GGU GGG CGC CGU AAU CUG CUU CGG CAG AGG GCG GGU GGG UGG ACG GCG CAU CGU AAU UGU AGC UGA UGC UGA CG |
| Honeydew 5′ -20 nt | GGG AGA GAA GGC GAU CUG GUG GGC GCC GUA AUC UGC UUC GGC AGA GGG CGG GUG GGU GGA CGG CGC AUC GUA AUU GUA GCU GAU GCU GAC G |
| Honeydew 5′ -30 nt | GGG AGA GAG UGG GCG CCG UAA UCU GCU UCG GCA GAG GGC GGG UGG GUG GAC GGC GCA UCG UAA UUG UAG CUG AUG CUG ACG |
| Honeydew 5′ -40 nt | GGG AGA GAU AAU CUG CUU CGG CAG AGG GCG GGU GGG UGG ACG GCG CAU CGU AAU UGU AGC UGA UGC UGA CG |
| Honeydew 5′ -50 nt | GGG AGA GAC GGC AGA GGG CGG GUG GGU GGA CGG CGC AUC GUA AUU GUA GCU GAU GCU GAC G |
| Honeydew 3′ -10 nt | GGG AGA GAU AAA CUG GAC CAC GGU GAC AAG GCG AUC UGG UGG GCG CCG UAA UCU GCU UCG GCA GAG GGC GGG UGG GUG GAC GGC GCA UCG UAA UUG UAG CU |
| Honeydew 3′ -20 nt | GGG AGA GAU AAA CUG GAC CAC GGU GAC AAG GCG AUC UGG UGG GCG CCG UAA UCU GCU UCG GCA GAG GGC GGG UGG GUG GAC GGC GCA UCG U |
| Honeydew 3′ -30 nt | GGG AGA GAU AAA CUG GAC CAC GGU GAC AAG GCG AUC UGG UGG GCG CCG UAA UCU GCU UCG GCA GAG GGC GGG UGG GUG GAC |
| Honeydew 3′ -40 nt | GGG AGA GAU AAA CUG GAC CAC GGU GAC AAG GCG AUC UGG UGG GCG CCG UAA UCU GCU UCG GCA GAG GGC GG |
| Honeydew 3′ -50 nt | GGG AGA GAU AAA CUG GAC CAC GGU GAC AAG GCG AUC UGG UGG GCG CCG UAA UCU GCU UCG G |
| Honeydew 5′ -20 nt, 3′ -20 nt | GGG AGA GAA GGC GAU CUG GUG GGC GCC GUA AUC UGC UUC GGC AGA GGG CGG GUG GGU GGA CGG CGC AUC GU |
| Honeydew 5′ -30 nt, 3′ -20 nt | GGG AGA GAG UGG GCG CCG UAA UCU GCU UCG GCA GAG GGC GGG UGG GUG GAC GGC GCA UCG U |
| Honeydew 5′ -20 nt, 3′ -30 nt | GGG AGA GAA GGC GAU CUG GUG GGC GCC GUA AUC UGC UUC GGC AGA GGG CGG GUG GGU GGA C |
| Honeydew 5′ -30 nt, 3′ -30 nt | GGG AGA GAG UGG GCG CCG UAA UCU GCU UCG GCA GAG GGC GGG UGG GUG GAC |
| R8-04 | GGG AGA GAU AAA CUG GAC CAC GGU GAC UGA UGU UGG GUG AAC GCA CUG UCG CCU GCU UCG GCA GCC AGA CUU UUU UGA UGG ACC GUG CUG UAA UUG UAG CUG AUG CUG ACG |
| R8-05 | GGG AGA GAU AAA CUG GAC CAC GGU GAC AGU CAG GCU AGU UAG GCG ACA GAU UCU GCU UCG GCA GAA GGG CGG UAU GUG GGU GGU GUC GUU AAU UGU AGC UGA UGC UGA CG |
| R8-06 | GGG AGA GAU AAA CUG GAC CAC GGU GAC GUG CUU GCA GGC GGU GGG CGU CGG UCU GCU UCG GCA GUG UGU AUU GGU UUG GCG CGG UGG GGC UAA UUG UAG CUG AUG CUG ACG |
| R8-07 | GGG AGA GAU AAA CUG GAC CAC GGU GAC UGG GUG AUG CGG AGA UGC CGG GAA GCU GCU UCG GCA GGA CGU CGU UUG GCG UUG GUG AGC GCA UAA UUG UAG CUG AUG CUG ACG |
| R8-08 | GGG AGA GAU AAA CUG GAC CAC GGU GAC AUG ACG CCU CAA CGC GCU UAU CUU UCU GCU UCG GCA GGU CUU CCG CGU UCG GUA AUU GUA GCU GAU GCU GAC G |
| R8-09 | GGG AGA GAU AAA CUG GAC CAC GGU GAC GCC UGC GUA GCG GUU UGU AGU CGG CUG CUU CGG |

Formatted Table

Formatted Table

| | |
|---|---|
| | CAG GAA CAU GGU UGG GCG UAA UUG UAG CUG AUG CUG ACG |
| R8-10 | GGG AGA GAU AAA CUG GAC CAC GGU GAC ACA GUC UGC GGG CCG AGU GGU GAA GCU GCU UCG GCA GGU UUG GCG UUG GGU GCG UGG GUG GCU UAA UUG UAG CUG AUG CUG ACG |
| Forward Primer | AAT TCT AAT ACG ACT CAC TAT AGG GAG AGA TAA ACT GGA CCA CGG TGA C |
| Reverse Primer | CGT CAG CAT CAG CTA CAA TT |
| nsRNA (non selected) | GGG AGA GAU AAA CUG GAC CAC GGU GAC CAC CAG AUC CUC AUA GUC GUC UCA CCA GAU CCU CAU AGU CGU CUC ACC AGA UCA AUU GUA GCU GAU GCU GAC G |

**Supplementary Table 1: Oligonucleotides used.** R0-Library Template originally ordered from IDT and amplified in-house for selection. Ten sequences, R8-01 through R8-10, ordered after eight rounds of selection for characterization. Forward amplification primer used each round for PCR that installs the T7 transcription promoter. Reverse primer used for reverse transcription and PCR amplification. An RNA sequence, nsRNA, that was not a member of the selection library and did not undergo selection, previously screened and known not to enhance conditional fluorescence of tartrazine.

## Supplementary Methods

### Viscosity and solvent effects on tartrazine

Tartrazine (10 µM) was dissolved in either water or 90% glycerol, ethanol, methanol, or acetonitrile. Samples were imaged using an Aplegen Omega Lum G system with blue epi-illumination (460-490 nm) and orange emission filter according to the SYBR imaging mode. Exposure and image processing settings were held constant for the set of images taken at room temperature and subsequently for images of the frozen samples after flash freezing in liquid nitrogen.

### Sequencing analysis criteria

After each round of selection, the enriched library was submitted for ~~Illumina based AMPLICON-EZ~~ high-throughput sequencing as described in the methods. Sequencing data ~~was~~were trimmed to remove sequencing adapters, filtered for read quality, and counted as described in the methods in the main text. From this count file various criteria were investigated to probe the selection for sequences of interest. Using an in-house HoneydewCountCompiler.py script, sequences were assembled into a spreadsheet capable of sorting by rank, reads, or RPM per round, by total RPM, by average enrichment per round, or by total enrichment. FASTAptamer count files were also used for FASTAptamer clustering and motif search analysis.

**Formatted:** Space Before: 0 pt, After: 0 pt

The HoneydewCountCompiler sorting was used to find sequences of interest based upon RPM and enrichment, while other sequences of interest were based on the FASTAptamer clustering and motif searches. Criteria we weighted with heavy importance were clustering in rounds 7 and 8, enrichment of individual sequences between rounds 7 and 8 specifically, total enrichment of individual sequences across rounds 0 through 8, total cumulative RPM of individual sequences

**Formatted:** Indent: First line: 0"

from rounds 0 through 8, and finally (the criteria that was most indicative of fluorescence) RPM of individual sequences for round 8 only.

## HoneydewCountCompiler.py script

This script was generated with input from the following large language models: `GPT-3.5`, `GPT-3.5-Turbo`, `GPT-4o`, `GPT-4o-mini`, `gemini-2.5-flash`, `gemini-2.5-pro`, `gemini-3.0-flash`, `gemini-3.0-pro`, `gemini-3.1-flash`, and `gemini-3.1-pro`. The final script and all outputs were manually reviewed by the authors.

**Formatted:** Font: Courier New

This script carries out sequence compilation and ~~levenshtein~~Levenshtein distance (LED) analysis. The script is designed to process and compile high-throughput sequencing data from multiple rounds of a selection. It filters the number of output sequences based on a minimum Reads Per Million (RPM) cutoff provided by the user or enforced by the script to allow a maximum of 1,048,575 sequences as that is the maximum number of rows permitted in an Excel workbook document. For each output sequence and each round of selection the script provides the round rank, round read, round RPM, average enrichment per round, total enrichment, and LED to provided sequences of interest if applicable. For this Honeydew library design the script also calculates separate LED analysis of the ~~5’~~5′ and ~~3’~~3′ randomized regions separated by the defined interior constant region to probe for hybridizations between sequences of interest.

HoneydewCountCompiler.py requires Python 3.x with the following packages: `numpy`, `pandas`, and `openpyxl`. The script uses the following input files: an input .fasta file of counted sequences for each round of selection (naming convention “R#_count.fasta” for each round of selection), and an Excel spreadsheet .xlsx file for known sequences LED analysis. The .fasta count files in this work were assembled using FASTAptamer 2 Count which assembles the sequencing reads from a round into a ~~.fasta~~FASTA file formatted with sequence headers “>Rank-Reads-RPM” on the first line, followed by the sequence on the next line. The known sequences .xlsx file must have a header row 1 containing the following data: Column A- blank, Column B- NickName (the ID or name of each known sequence), Column C- Sequence (the full known sequence), Column

**Formatted:** Font: Courier New

**Formatted:** Font: Courier New

D- 5primehalf (the 5’5′ random region of the known sequence), Column E- 3primehalf (the 3’3′ random region of the known sequence), Row 2 Column M- this cell specifically must contain the string sequence of the interior constant region of the designed library as the script uses this string to separate the output sequences into 5’5′ and 3’3′ halves for LED comparison.

The script can either be run in interactive mode with no arguments, providing step-by-step prompts for all necessary inputs, or the prompts can be bypassed by providing the arguments directly in the terminal. The arguments are in the following order: > python script.py [Number of files] [File1.fasta] ... [FileN.fasta] [Optional: RPM Cutoff] [Round Range] [Known.xlsx]. The two output files generated will be a .tsv data file that can be opened in Excel, and a .txt report file briefly detailing the requested RPM cutoff, the enforced RPM cutoff, how many sequences were filtered out, and the script execution time. The output columns of the data files are as follows: Column A- Sequence (the sequence extracted from the .fasta count files), Column B- Length (total nucleotides in the sequence), Column C- Rank RX (the abundance rank of the sequence in round X, extracted from the .fasta count files headers), Column D- Reads RX (the raw read count of the sequence in round X), Column E- RPM RX (the reads per million of the sequence in round X), Column F- Total RPM (the sum of the RPM values for this sequence across all analyzed rounds), Column G- RPM Enrichment (The average fold-change of this sequence across all successive rounds; the script calculates the ratio of $\text{RPM}_{(\text{later round})}$ / $\text{RPM}_{(\text{earlier round})}$ for every possible pair of rounds, and averages them, which favors sequences that enriched quickly over only a few rounds and didn’t occur in any earlier rounds), Column H- Total Enrich (the sum total of enrichment without averaging for the number of round pairs; this column will lend favor to sequences that persisted throughout many selection rounds), Column I- LED (the minimum Levenshtein distance between the full experimental sequence and the closest matching full sequence in the provided known

sequences file), Column J- ID (the NickName of the known sequence with the reported lowest LED), Column K- ~~5’~~5′ LED (the minimum Levenshtein distance between ~~5’~~5′ region of the experimental sequence and the closest matching ~~5’~~5′ region in the known sequences file), Column L- ~~5’~~5′ ID (the NickName of the known sequence with the ~~5’~~5′ region that resulted in the lowest ~~5’~~5′ LED), Column M- ~~3’~~3′ LED (the minimum Levenshtein distance between ~~3’~~3′ region of the experimental sequence and the closest matching ~~3’~~3′ region in the known sequences file), Column N- ~~3’~~3′ ID (the NickName of the known sequence with the ~~3’~~3′ region that resulted in the lowest ~~3’~~3′ LED).

If the user does not have known sequences of interest, or wants to forgo the LED analysis, simply do not provide a known sequences input file and the LED analysis columns in the output data file will be left blank. If the library of interest does not have ~~5’~~5′ and ~~3’~~3′ random regions separated by a defined interior constant region, leave cell M2 of the known sequences file blank.

## Honeydew truncation sequence transcription reactions

Two sets of truncated Honeydew DNA templates were ordered for synthesis and screened for fluorescence activation of tartrazine via *in vitro* transcription reactions. All DNA templates were prepared as synthetic oligonucleotides from Integrated DNA Technologies (Coralville, IA) with singular 5′ or 3′ truncations ordered as eBlock Gene Fragments while paired 5′/3′ doubly truncated sequences were ordered as ssDNA Oligos and annealed in-house. Transcription reactions containing 100 μM tartrazine were monitored for real-time fluorescence enhancement via SpectraMax Gemini EM microplate reader (Molecular Devices; SoftMax Pro 5.4).

## Fluorescence binding kinetics

Baseline fluorescence of 2.5 µM tartrazine in fluorescence assay buffer was recorded for 30 seconds using a Cary Eclipse fluorescence spectrophotometer (Agilent Technologies) with settings: excitation 420 nm, excitation and emission slits 10 nm, PMT 950 V, and 0.2 second averaging time per data point. After 30 seconds, the cuvette was briefly removed from the light path (~4 seconds) to pipette mix pre-folded Honeydew aptamer to a final concentration of 5 µM. Fluorescence monitoring then continued for a total recorded period of ten minutes.

**Formatted:** Indent: First line: 0"
